# A supramolecular Tröger's base derived coordination zinc polymer for fluorescent sensing of phenolic-nitroaromatic explosives in water†
†Electronic supplementary information (ESI) available: Spectroscopic (multinuclear NMR, IR HRMS) characterizations, optimized structure, TGA, powder diffraction and fluorescence titration profiles. See DOI: 10.1039/c6sc04367d


**DOI:** 10.1039/c6sc04367d

**Published:** 2016-10-25

**Authors:** Sankarasekaran Shanmugaraju, Charlyne Dabadie, Kevin Byrne, Aramballi J. Savyasachi, Deivasigamani Umadevi, Wolfgang Schmitt, Jonathan A. Kitchen, Thorfinnur Gunnlaugsson

**Affiliations:** a School of Chemistry and Trinity Biomedical Sciences Institute (TBSI) , Trinity College Dublin , The University of Dublin , Dublin 2 , Ireland . Email: shanmugs@tcd.ie ; Email: gunnlaut@tcd.ie; b School of Chemistry and Centre for Research on Adaptive Nanostructures and Nanodevices (CRANN) , Trinity College Dublin , The University of Dublin , Dublin 2 , Ireland; c Chemistry, Faculty of Natural and Environmental Sciences , University of Southampton-Highfield , Southampton , SO17 1BJ , UK

## Abstract

A V-Shaped 4-amino-1,8-napthalimide derived tetracarboxylic acid linker (**L**; bis-[*N*-(1,3-benzenedicarboxylic acid)]-9,18-methano-1,8-naphthalimide-[*b*,*f*][1,5]diazocine) comprising the Tröger's base (TB) structural motif was rationally designed and synthesised to access a nitrogen-rich fluorescent supramolecular coordination polymer. By adopting the straight forward precipitation method, a new luminescent nanoscale Zn(ii) coordination polymer (**TB-Zn-CP**) was synthesized in quantitative yield using Zn(OAc)_2_·2H_2_O and tetraacid linker **L** (1 : 0.5) in DMF at room temperature. The phase-purity of as-synthesised **TB-Zn-CP** was confirmed by X-ray powder diffraction analysis, infra-red spectroscopy, and elemental analysis. Thermogravimetric analysis suggests that **TB-Zn-CP** is thermally stable up to 330 °C and the morphological features of **TB-Zn-CP** was analysed by SEM and AFM techniques. The N_2_ adsorption isotherm of thermally activated **TB-Zn-CP** at 77 K revealed a type-II reversible adsorption isotherm and the calculated Brunauer–Emmett–Teller (BET) surface area was found to be 72 m^2^ g^–1^. Furthermore, **TB-Zn-CP** displayed an excellent CO_2_ uptake capacity of 76 mg g^–1^ at 273 K and good adsorption selectivity for CO_2_ over N_2_ and H_2_. The aqueous suspension of as-synthesized **TB-Zn-CP** showed strong green fluorescence (*λ*_max_ = 520 nm) characteristics due to the internal-charge transfer (ICT) transition and was used as a fluorescent sensor for the discriminative sensing of nitroaromatic explosives. The aqueous suspension of **TB-Zn-CP** showed the largest quenching responses with high selectivity for phenolic-nitroaromatics (4-NP, 2,4-DNP and PA) even in the concurrent presence of other potentially competing nitroaromatic analytes. The fluorescence titration studies also provide evidence that **TB-Zn-CP** detects picric acid as low as the parts per billion (26.3 ppb) range. Furthermore, the observed fluorescence quenching responses of **TB-Zn-CP** towards picric acid were highly reversible. The highly selective fluorescence quenching responses including the reversible detection efficiency make the nanoscale coordination polymer **TB-Zn-CP** a potential material for the discriminative fluorescent sensing of nitroaromatic explosives.

## Abstract 2

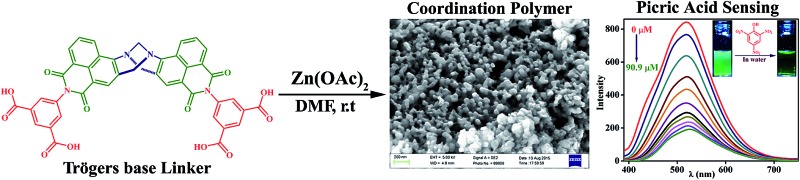
A Tröger’s base functionalized luminescent nanoscale Zn(II) coordination polymer (**TB-Zn-CP**) is synthesized and used as selective fluorescence sensor for phenolic nitroaromatics in water.

## Introduction

Supramolecular self-assembly chemistry provides a fascinating platform for developing functional structures and materials that can have applications in various fields.^1^ Discovery of suitable chemical sensors and sensor materials for the rapid and selective detection of chemical explosives at trace levels has attracted significant attention in recent times.^2^ The reliable identification of chemical explosives in post-blast detritus is of great importance for forensic analysis and criminal investigations, as well as in an effort to combat terrorism, improve national security and control environmental pollution, *etc.* ^2^ Among the various known high energetic chemical substances of such nature are the nitroaromatic compounds (NACs). These include picric acid (PA), 2,4,6-trinitrotoluene (TNT), 2,4-dinitrotoluene (2,4-DNT) and 2,6-dinitrotoluene (2,6-DNT); all of which are used as primary components in several known chemical explosives owing to their low cost syntheses and easy transportation.^2b,3^ Several examples of supramolecular sensory systems for the detection of such explosives have been developed in recent times.^2–5^ Apart from its explosive nature, PA is also used on a small scale for various medical formulations and as an antiseptic agent and also finds application as yellow pigment in dye/leather industries.^6^ Because of its multifarious uses and high water solubility (∼14 g L^–1^ at 20 °C), PA and its derivatives have become a major environmental pollutant and severely affect soil and ground water causing serious health hazards.^7^ According to the World Health Organization (WHO), the allowed concentration of PA in ground water is 0.001 mg L^–1^ and above this level it is considered as toxic to living organisms.^8^ Short time exposure to PA residues induce eye irritation, headache, anemia and skin related diseases while long time exposure may lead to kidney problems and severe liver and respiratory organ damage.^6a,9^ Therefore, the development of selective and highly sensitive sensors with a fast-response time for trace and discriminative detection of PA from other nitroaromatic explosives is vital for environmental remediation, civilian safety, and for military operations.^5b,10^

Currently, a wide range of sophisticated techniques like metal detectors, canine teams, ion-mobility spectrometry (IMS), surface-enhanced Raman spectroscopy (SERS), gas chromatography (GC), X-ray diffraction and cyclic voltammetry *etc.* are available for the detection of nitroaromatic explosives.^11^ But the routine-field analysis using these techniques is often limited due to their high cost, lack of portability, low selectivity and difficulty to operate on-site. However, in recent years, fluorescence detection techniques have become an effective and alternative detection method for nitroaromatic explosives owing to their high sensitivity, easy visualisation, portability and fast response time for detection.^12^ In fluorescence-based sensing, the emission characteristic of the fluorophores is perturbed upon binding with analytes.^13^ The presence of electron withdrawing nitro (–NO_2_) groups makes the nitroaromatics electron-deficient and thus they form effective charge-transfer complexes with electron-rich fluorophores by subsequent fluorescence quenching.^5a,14^ In light of this, until recently, a wide variety of fluorescent sensors have been designed and successfully employed for NACs detection.^1b,3b,15^

One such material that has received immense interest, in recent years, for NACs detection is luminescent metal–organic frameworks (MOFs) or coordination polymers (CPs) on account of their easy synthesis, unique and functional properties, permanent porosity, high thermal/chemical stability, systematically tuneable electronic structures, intriguing structural diversity, good recyclability, and so on.^1a,5a,b,7a,13b,16^ In contrast to the conventional CPs, the miniaturized coordination polymers can have entirely different size-dependent physico-chemical properties.^17^ Scaling down the particle size of bulk crystalline CPs to their micro-/nanoscale regime is expected to facilitate their easy dispersion in aqueous media and increases the interaction with analytes and thus enhances the sensing ability.^18^ To the best of our knowledge, only a limited number of luminescent nanoscale coordination polymers have been developed and exploited as potential fluorescence sensors for the detections of NACs. For example, Maji *et al.* have synthesised a rod-like Gd(iii) coordination polymer from oligo-(*p*-phenylene-ethynylene)dicarboxylic acid and demonstrated its sensing ability towards NACs in ethanol.^19^ Zhang *et al.* reported a fluorescent nanoscale Zn(ii)–anthracene coordination polymer with cubic morphology which exhibited efficient sensing characteristics for NACs vapour,^20^ while Qiu *et al.* described the nitroaromatic sensing characteristics of Cd(ii) based coordination polymer tubular nanotubes in the solid-state.^21^ Huang *et al.* synthesized a π-system based fluorescent Tb(iii)-coordination polymer hollow nanospheres dispersed in DMF showing good selectivity and sensitivity for sensing NACs.^18b,22^ However, the nitroaromatic sensing characteristics of these and other already reported nanoscale CPs involve sensing in a pure organic medium or in a vapour phase. For the practical applicability, the sensing of NACs in aqueous media is highly desirable, particularly due to its environmental significance.^15e,23^ Moreover, and somewhat surprising, there are very few reports on nanoscale CP based sensor materials having discriminative sensing abilities for phenolic-nitroaromatics in aqueous medium.^5b,10b,15e,16a^ One possible way to increase the selectivity of CPs is to functionalise them with Lewis base (amine and pyridine) functional groups that could selectively recognize phenolic-nitroaromatics through intermolecular H-bonding interactions between Lewis bases and the hydroxy group of phenolic-nitroaromatics.^5b,15e,24^ 
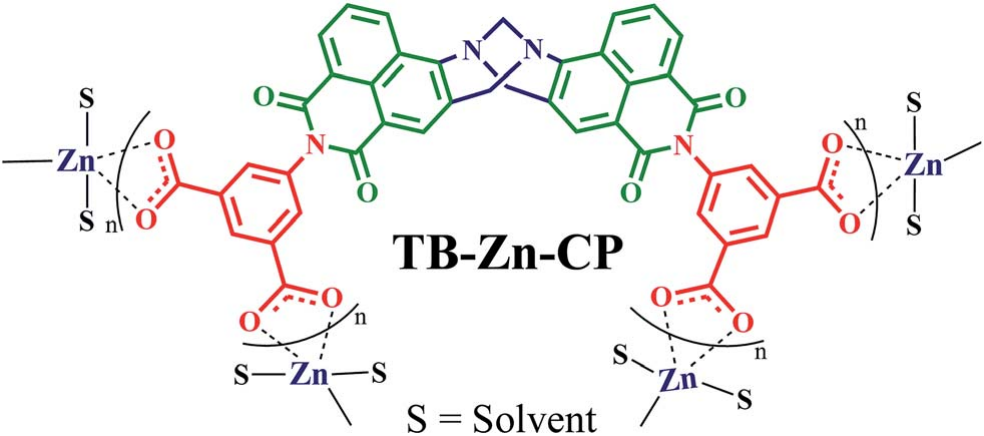


Based on this consideration, we rationally designed and synthesized a novel fluorescent nanoscale Zn(ii)-coordination polymer functionalized with a Tröger's base structural motif, **TB-Zn-CP**, from the 4-amino-1,8-napthalimide derived tetracarboxylic acid linker (**L**) as Tröger's bases based on this motif have been shown to be excellent supramolecular building blocks in our laboratory.^26^ The intrinsic chirality of the Tröger's base moiety favours the unique V-shaped arrangement of two 1,8-naphthalimides (as we have shown by using X-ray crystallography) facilitating the guest binding through multiple supramolecular interactions and subsequently demonstrating superior sensing capability for analyte detection. We foresaw that the Lewis base characteristics of the Tröger's base would increase the selectivity of **TB-Zn-CP** for the discriminative detection of phenolic nitroaromatics through intermolecular H-bonding interactions.^23*b*^ As we expected, **TB-Zn-CP** showed highly sensitive and discriminative sensing ability for the rapid detection of phenolic nitroaromatics such as PA, 2,4-DNT, 4-NP, even in the presence of other potential competing NACs. As it has also recently been shown by McKeown and co-workers, that Tröger's base containing (covalently linked) polymers can be used effectively for gas-uptake and storage of gases,^25^ we also investigated these properties for **TB-Zn-CP**. Indeed, **TB-Zn-CP** displayed an excellent CO_2_ gas uptake capacity of 76 mg g^–1^ at 273 K and good adsorption selectivity for CO_2_ over other gases such as N_2_ and H_2_, furthering the application of this type of CPs.

## Results and discussion

### Synthesis and characterisation of tetracarboxylic acid linker (**L**)

**Scheme 1:**
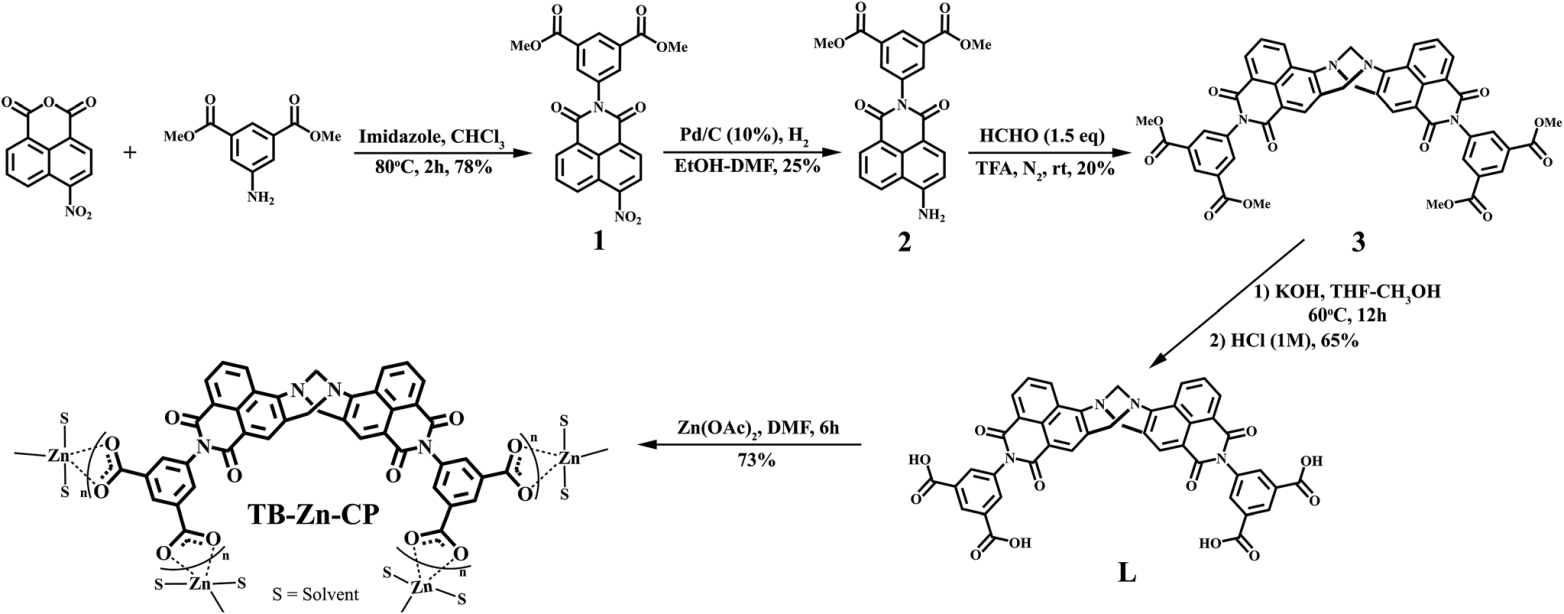
Synthesis of the bis-naphthalimide derived Tröger’s base tetracarboxylic acid linker (**L**) from commercially available 4-nitro-1,8-naphthalic anhydride, that was then used to generate the Zn(ii) coordination polymer (**TB-Zn-CP**).

The 4-amino-1,8-naphthalimide containing Tröger's base tetracarboxylic acid linker (**L**; bis-[*N*-(1,3-benzenedicarboxylic acid)]-9,18-methano-1,8-naphthalimide-[*b*,*f*][1,5]diazocine) was prepared as a racemic mixture in four steps from the commercially available 4-nitro-1,8-naphthalic anhydride as shown in Scheme 1.

4-Nitro-1,8-naphthalic anhydride and dimethyl-5-aminoisophthalate were refluxed at 80 °C in CHCl_3_ along with imidazole for 2 hours giving rise to the formation of the 4-nitro-1,8-naphthalimide (**1**) precursor. The reduction of **1** by catalytic hydrogenation with 10% Pd/C at 3 atm of H_2_ in ethanol/dimethylformamide gave the corresponding 4-amino-1,8-naphthalimide **2**. Compound **2** was next converted into the Tröger's base, using a method developed in our laboratory,^26^ involving the use of 1.5 equiv. of paraformaldehyde in neat trifluoroacetic acid under an inert atmosphere over 12 hours at room temperature. The resulting reaction mixture was basified (pH > 10) using aq. NH_3_ and followed by extraction with CH_2_Cl_2_ which gave tetra ester derivative **3** in 20% yield after trituration with cold diethyl ether. Subsequent hydrolysis of **3** under basic conditions led to the isolation of target tetracarboxylic acid linker **L** as a bright orange solid in 65% yield.

The formation of **L** was fully characterized by various spectroscopic techniques (see Experimental section and ESI†) such as IR, multinuclear (^1^H and ^13^C) NMR and HRMS. The ^1^H NMR spectrum confirmed the identity of **L** by the presence of a well-separated doublet of doublet peaks in between 5.24 and 4.70 ppm corresponding to the methylene (–CH_2_N) protons of the diazocine moiety and also reflecting the *C*_2_ symmetry of **L** (Fig. S7, ESI†).^26^ The high resolution mass spectrometry (HRMS) analysis of **L** showed two sharp peaks at *m*/*z* = 787.1308 and 393.0595 corresponding to the molecular ions [M – H]^–^ and [M – 2H]^2–^, respectively (Fig. S12, ESI†). The IR spectrum of **L** showed several intense peaks at *ν* = 3350 cm^–1^, 1705 cm^–1^ and 1239 cm^–1^ accounting for –OH, C=O and C–N stretching, respectively which indicated the presence of –COOH and Tröger's base functional groups (Fig. S16, ESI†).^27^ In view to gain further insight into the molecular structure of **L**, the energy minimized structure was obtained using density functional theory (DFT) calculation.‡
‡M. J. Frisch, G. W. Trucks, H. B. Schlegel, G. E. Scuseria, M. A. Robb, J. R. Cheeseman, G. Scalmani, V. Barone, B. Mennucci, G. A. Petersson, H. Nakatsuji, M. Caricato, X. Li, H. P. Hratchian, A. F. Izmaylov, J. Bloino, G. Zheng, J. L. Sonnenberg, M. Hada, M. Ehara, K. Toyota, R. Fukuda, J. Hasegawa, M. Ishida, T. Nakajima, Y. Honda, O. Kitao, H. Nakai, T. Vreven, J. A. Montgomery, J. E. Peralta, F. Ogliaro, M. Bearpark, J. J. Heyd, E. Brothers, K. N. Kudin, V. N. Staroverov, R. Kobayashi, J. Normand, K. Raghavachari, A. Rendell, J. C. Burant, S. S. Iyengar, J. Tomasi, M. Cossi, N. Rega, J. M. Millam, M. Klene, J. E. Knox, J. B. Cross, V. Bakken, C. Adamo, J. Jaramillo, R. Gomperts, R. E. Stratmann, O. Yazyev, A. J. Austin, R. Cammi, C. Pomelli, J. W. Ochterski, R. L. Martin, K. Morokuma, V. G. Zakrzewski, G. A. Voth, P. Salvador, J. J. Dannenberg, S. Dapprich, A. D. Daniels, Farkas, J. B. Foresman, J. V. Ortiz, J. Cioslowski and D. J. Fox, Wallingford CT, 2009. The structure has been optimized at M062X/6-31G* level and optimized structure along with the HOMO and LUMO orbitals are given in Fig S17 and S18 (see ESI†). The energy minimized structure of **L** suggests that the methao-1,5-diazocine ring with an angle of 95.40° between the two 1,8-naphthalimide moieties gives rise to the unique V-shaped geometry. It is also evident that the electron density is more localized near the Tröger's base regions and hence the electron-deficient analyte is expected to show preferential binding affinity with Tröger's base moiety. This angle corresponds well with that obtained from crystal structure analysis of a series of 4-amino-1,8-naphthalimide based Tröger's bases developed in our laboratory.^26*c*^

### Synthesis and characterization of nanoscale Zn(ii) coordination polymer **TB-Zn-CP**

In a typical synthesis, the nanoscale coordination polymer **TB-Zn-CP** was prepared by stirring Zn(OAc)_2_·2H_2_O (1 eq.) and tetracarboxylic acid linker **L** (0.5 eq.) in DMF solution (4 mL) at room temperature for 6 hours. While several examples of Zn(ii) targeted naphthalimide structures have been developed to date, no such naphthalimide Tröger's base ligands have been developed.^28^ The coordination of Zn(ii) ions to the carboxylate groups of the Tröger's base was evidenced from the instantaneous formation of orange colloidal suspensions; which was collected from the reaction mixture by centrifugation, the resulting residue being washed with fresh DMF *via* three successive dispersion cycles. The chemical composition of **TB-Zn-CP** was determined through elemental analysis, and it was found to be consistent with a molecular formula of Zn**L**·2H_2_O·DMF, in which the deprotonated linker (**L**–4H) is interconnected by Zn(ii) metallic nodes through metal–ligand coordinations. The product was further characterised by using FT-IR spectroscopy, the result showing a strong transitions at 1573 cm^–1^ (symmetrical) and 1402 cm^–1^ (asymmetrical), corresponding to the coordinated carboxylate (–COO^–^) stretching frequencies (Fig. S19, ESI†).^29^ A strong band at 1657 cm^–1^ was assigned to C=O stretching vibration of entrapped DMF solvent molecules, while a broad band, with the maximum at 3449 cm^–1^, was assigned to hydrogen bonded H_2_O molecules (Fig. S19, ESI†).

**Fig. 1:**
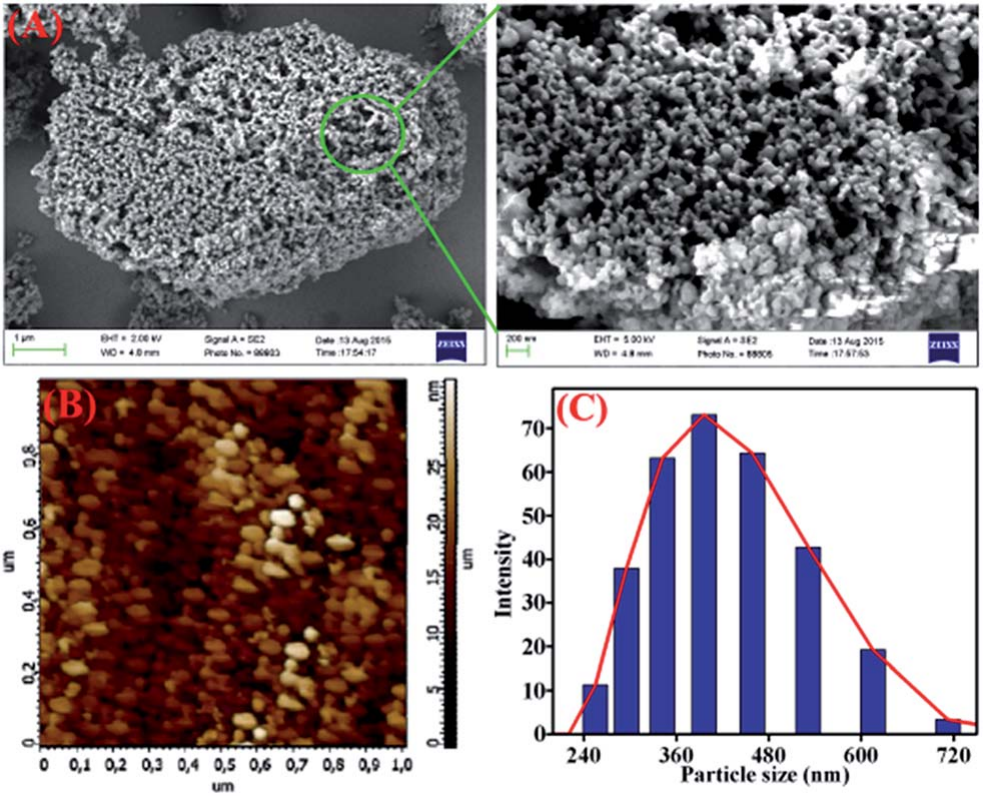
(A) Scanning electron microscopy and atomic force microscopy images (B) of as-synthesized **TB-Zn-CP**. (C) Dynamic light scattering (DLS) measurements of **TB-Zn-CP** dispersed in water.

**TB-Zn-CP** was shown to be amorphous as indicated by X-ray powder diffraction measurement (Fig. S20, ESI†). The TGA analysis of **TB-Zn-CP** under nitrogen atmosphere showed an initial weight loss of ∼6% at 120 °C due to the loss of coordinated/trapped solvent molecules and the desolvated material was stable up to 330 °C and retains ∼24% of its initial mass at 500 °C (Fig. S21, ESI†). To comprehend the morphology of **TB-Zn-CP**, we performed Scanning Electron Microscopy (SEM) and atomic-force microscopy (AFM) analysis of the product. As shown in Fig. 1, the SEM (Fig. 1A) and AFM (Fig. 1B) images of **TB-Zn-CP** showed polydispersed spherical particles that were further aggregated to give a nanoporous structure. The energy-dispersive X-ray spectroscopy (EDX) analysis of **TB-Zn-CP** exhibited the signature peak for Zn, C, O and N (Fig. S22, ESI†). Quanta area mapping study illustrated the uniform distribution of Zn, C, O and N over the entire sample (Fig. S22, ESI†). Further analysis, using dynamic light scattering (DLS), of aqueous suspension of **TB-Zn-CP** indicated that the particle sizes are in the nanoscale range, as shown in Fig. 1C.

**Fig. 2:**
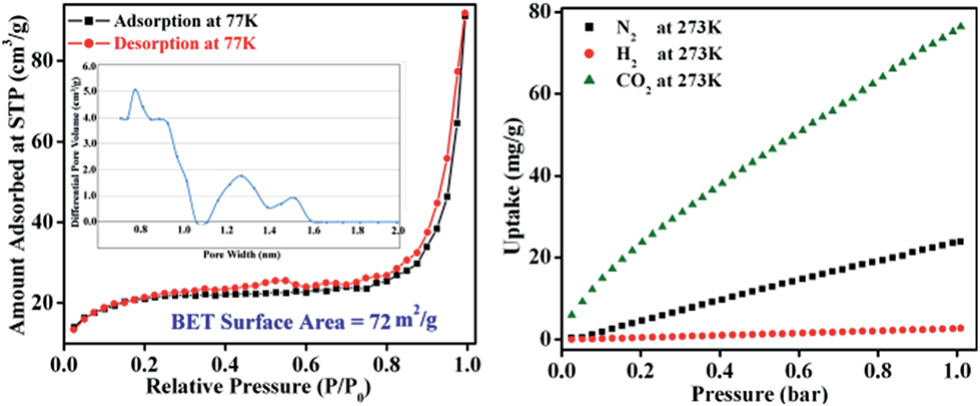
The N_2_ adsorption–desorption isotherm measured at 77 K (inset: corresponding pore-size distribution curve) of **TB-Zn-CP** (left). The uptake capacitates of **TB-Zn-CP** for CO_2_, N_2_ and H_2_ at 273 K (right).

The permanent porosity and surface area of **TB-Zn-CP** were verified by N_2_ adsorption isotherms on a Quantachrome AUTOSORP-IQ automated gas sorption analyser. The N_2_ adsorption isotherm at 77 K exhibits a conventional type-II reversible adsorption isotherm and the calculated Brunauer–Emmett–Teller (BET) surface area was found to be 72 m^2^ g^–1^, Fig. 2. The Non-Local Density Functional Theory (NLDFT) pore size distribution of **TB-Zn-CP** suggests the presence of porosity in the range of 0.7–1.5 nm as shown in Fig. 2. The presence of the CO_2_-philic nitrogen-rich Tröger's base unit encouraged us to also evaluate the CO_2_ capture properties. Hence, the **TB-Zn-CP** was activated under vacuum at 100 °C for 24 hours. The CO_2_ adsorption measurements of activated **TB-Zn-CP** up to 1 bar at 273 K showed an uptake of 76 mg g^–1^ (Fig. 2). The total uptake capacity of **TB-Zn-CP** for N_2_ and H_2_ at 273 K was lower which can be partially attributed to weak interactions with the nitrogen-rich Tröger's base moieties (*e.g.* Fig. 2). Selectivity for CO_2_ over N_2_ was calculated at 273 K by taking the linear part of each uptake slope from 0.025 to 0.178 bar and dividing the slopes which yielded a value of 2.6. The selectivity of **TB-Zn-CP** for CO_2_ over N_2_ can be ascribed to enhanced dipole–quadrupole interactions.^25a,30^

### Photophysical characteristics of **TB-Zn-CP** and **L**

**Fig. 3:**
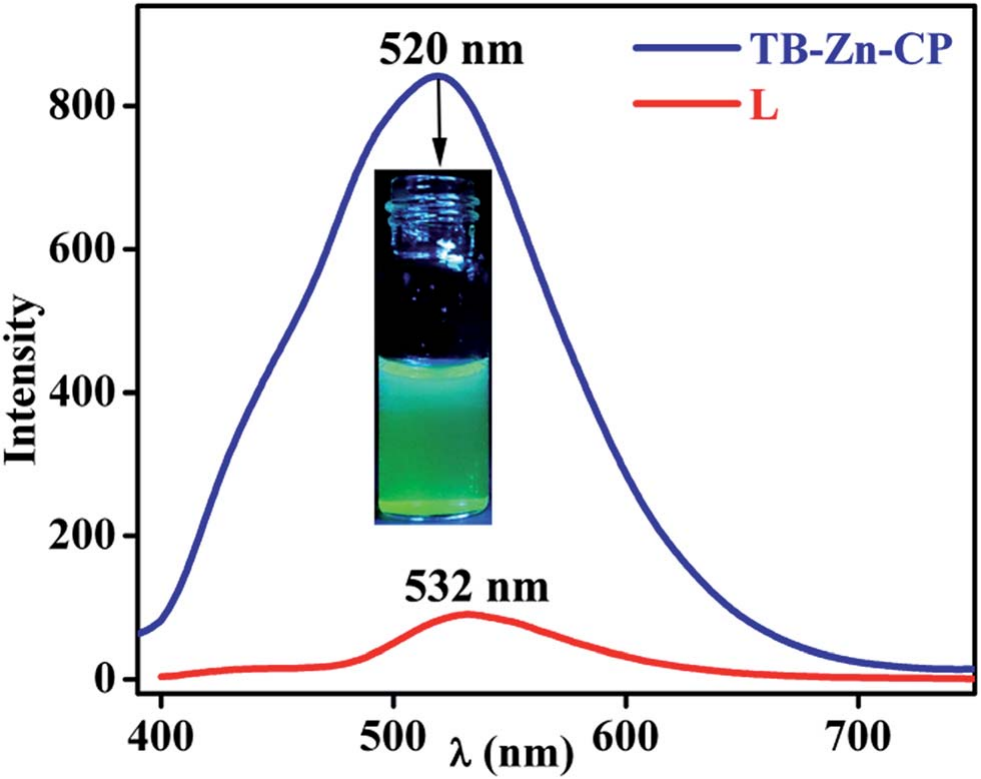
Fluorescence emission spectra of **TB-Zn-CP** (*λ*_max_ = 520 nm) and **L** (*λ*_max_ = 532 nm) dispersed in water (inset: visual color of **TB-Zn-CP** taken under UV lamp).

Coordination polymers based on electronically inert d^10^ metal ions and conjugated organic ligands, have been shown to often display strong photoluminescence characteristics, and as such, these can be employed as responsive materials, for instance, for applications in sensing.^15f,16a,31^ With this in mind, and given the nature of the Tröger's base structure, which we have previously shown to be an excellent candidate for the luminescent sensing of for instance, polyanions,^32^ we investigated the use of **TB-Zn-CP** in the sensing of NACs. Initially, the absorption and the fluorescence emission spectra of the Tröger's base **L** was recorded in de-ionised water. The absorption spectra (Fig. S23, ESI†) exhibited a typical 1,8-naphthalimide Tröger's base spectra, which was blue shifted by *ca.*, 60 nm compared to that seen for **2**, as the 4-amino-moiety of the Tröger's base is less conjugated, giving rise to a smaller internal charge transfer (ICT) character than that seen for **2**. This is in an agreement to that previously seen for other 1,8-naphthalimide Tröger's bases. Moreover, the fluorescence excitation and the emission spectra were recorded upon exciting at *λ* = 390 nm; the former structurally matching that seen in the absorption spectra. In the fluorescence emission spectra, a broad band centred at *λ* = 532 nm, Fig. 3, was observed, and this was assigned to the “push–pull” based ICT transition.^26,32^ Similarly, both the absorption and the emission spectra of the **TB-Zn-CP** colloidal particle were recorded dispersed in H_2_O. The results were similar to that seen above, except that the Zn(ii) coordination gave rise to a high energy absorption band in the absorption spectra; the ICT band of the naphthalimide Tröger's base appeared slightly blue shifted (Fig. S24, ESI†). Upon excitation at 380 nm, a very intense emission band was observed, between 400–700 nm, with *λ*_max_ = 520 nm, which was almost an order of magnitude more intense than seen for **L**, Fig. 3. This green emission was clear to the naked eye, even at low concentrations, as demonstrated in the inset in Fig. 3; the emission spectra of **TB-Zn-CP** was *ca.* 12 nm blue-shifted compared to **L**. The significant enhancement in the emission intensity (∼90%) of **TB-Zn-CP** can be ascribed to two main reasons. Firstly, long range electron communication between the adjacent fluorophores through Zn(ii) ions^10^ and secondly, due to through-space depression of photoinduced electron transfer (PET) quenching from the aryl tetracarboxylic acid moiety which is close to being orthogonal to the naphthalimide moiety. Here, upon coordination to Zn(ii), the oxidation potential of the receptor is enhanced, diminishing the thermodynamic pathway for PET to the naphthalimide centre, resulting in enhancement in naphthalimide emission. While such PET quenching is normally only seen for 4-amino-1,8-naphthalimide based structures, where the ion receptor is located, *via* a covalent spacer at the 4-amino moiety,^26a–d,33^ we have shown that upon incorporation of the diazocine moiety, the PET process for the resulting naphthalimide Tröger's base is modulated, allowing for such PET quenching to occur.^33^

### Discriminative detection of nitroaromatics explosives

As discussed above, the strong luminescence characteristics of **TB-Zn-CP** in aqueous medium was the driving force for evaluating the polymer as a fluorescent sensor for NACs. The following NAC analytes were chosen for the fluorescence sensing studies: 4-nitrotoluene (4-NT), 2,6-dinitrotoluene (2,6-DNT), 3-nitrotoluene (3-NT), 2,4-dinitrotoluene (2,4-DNT), nitrobenzene (NB), 2-nitrotoluene (2-NT), 3-nitrophenol (3-NP), 2-nitrophenol (2-NP), 4-nitrophenol (4-NP), 2,4-dinitrophenol (2,4-DNP) 2,4,6-trinitrotoluene (TNT) and picric acid (PA). To ensure complete solubility, stock solutions of all the analytes were prepared in a water–ethanol (9 : 1) solvent mixture and all titrations were preformed in triplicate. From a practical point of view, the detection of nitroaromatics in an aqueous medium is highly desirable and hence all the fluorescence titration experiments were performed in aqueous medium.

**Fig. 4:**
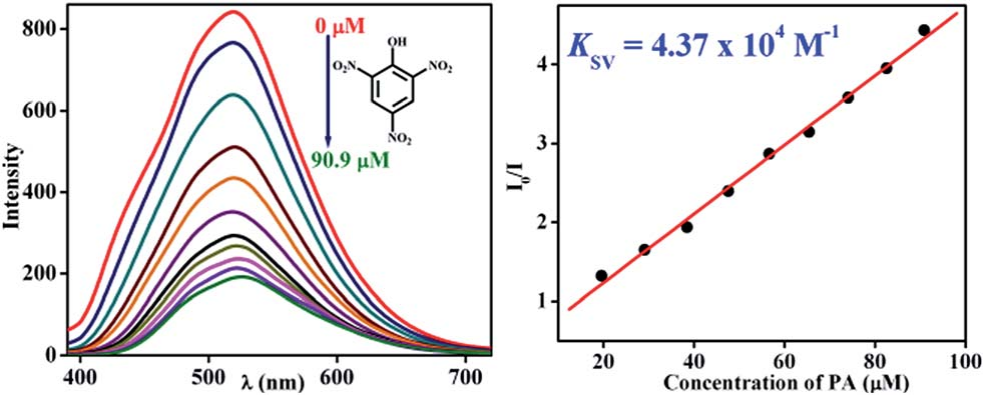
Observed fluorescence quenching (left) of **TB-Zn-CP** upon addition of PA (μM) in water and its corresponding Stern–Volmer plot (right).

To demonstrate the ability of **TB-Zn-CP** for the detection of nitroaromatic explosives, we first performed fluorescence titration experiments of **TB-Zn-CP** with PA as representative example of NACs. Concomitantly, the absorption spectra were recorded, but no significant changes were seen in the ICT band. In contrast, the gradual addition (20–200 μL) of 1 mM PA solution to an aqueous suspension of **TB-Zn-CP** elicited significant quenching in the fluorescence emission, as demonstrated in Fig. 4; with no other significant spectral changes (such as shifts in *λ*_max_) being observed. The **TB-Zn-CP** emission responses were analysed by fitting the data to a Stern–Volmer equation:(*I*_0_/*I*) = 1 + *K*_SV_[Q]where; *I*_0_ is the initial fluorescence intensity before the addition of analyte, *I* is the fluorescence intensity in the presence of analyte, [Q] is the molar concentration of analytes, and *K*_SV_ is the Stern–Volmer constant. The result for the PA titration is also shown in Fig. 4, where a linear Stern–Volmer plot was obtained from the fluorescence quenching titration profile, and the calculated Stern–Volmer constant *K*_SV_ was determined as 4.37 × 10^4^ M^–1^. This demonstrates both high binding affinity and a significant improvement over the current state of the art, as previously reported binding values for other Zn(ii)-based luminescent coordination polymers are usually determined in less competitive media or in organic solvents, which makes them significantly less useful for practical applications.^15f,16a,31,34^ The calculated higher rate of binding constant (*K*_SV_) is most likely due to the long-range electronic communication between the adjacent fluorophores upon Zn(ii)-coordination.^14b,16c,35^ The large spectral overlaps in the absorption spectrum of PA with the emission maximum of **TB-Zn-CP** (see Fig. S25, ESI†) confirms that an excited state energy transfer mechanism operates between **TB-Zn-CP** and PA.^15e,24b^ We propose that the reason for the fluorescence quenching is due to efficient charge-transfer (CT) complex formation between the electron-rich Tröger's base coordination polymer and electron-poor PA, preferably *via* hydrogen bonding.

**Fig. 5:**
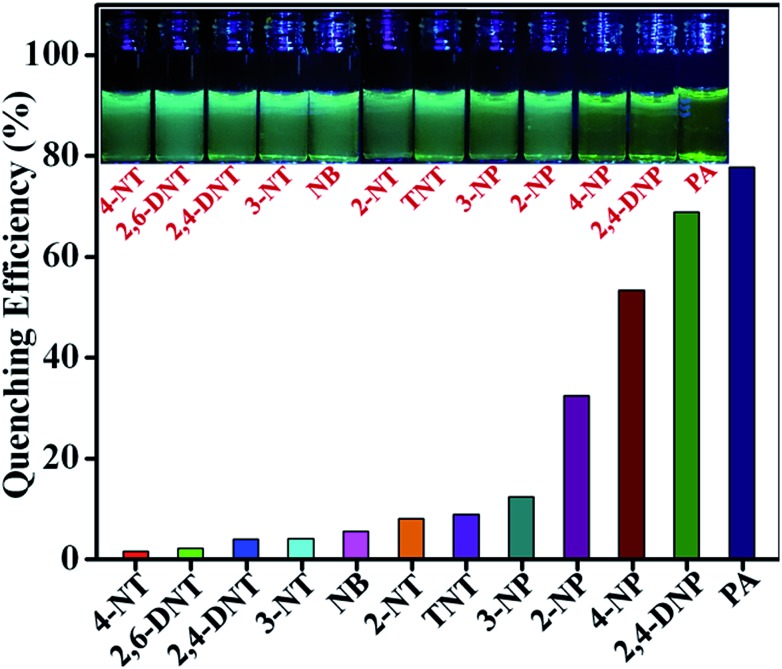
Extent of fluorescence quenching of **TB-Zn-CP** observed upon the addition of various analytes. Inset: the colour changes observed.

To explore the selectivity of **TB-Zn-CP** towards NACs, we performed similar fluorescence titrations, under identical spectroscopic conditions, with the other electron deficient NACs listed above. For these, **TB-Zn-CP** exhibited different fluorescence quenching responses, which allowed us to discriminate between structurally similar NACs. As shown in Fig. 5, the titrations with phenolic nitroaromatics such as PA, 2,4-DNP, 4-NP resulted in the highest quenching effect compared to other NACs; the changes also being clearly visible to the naked eye. Here, PA displayed the highest quenching response (78%) due to its more electron deficient nature because of the strong electron-withdrawing tendency of the substituted –NO_2_ groups. Notably, the non-phenolic NACs such as NB, 2-NT, 4-NT, 4-NT, 2,6-DNT, 2,4-DNT and TNT exhibited moderate to poor quenching effects (Fig S26–S35, ESI†). The descending order of quenching efficiencies are as follows: PA > 2,4-DNP > 4-NP > 2-NP > 3-NP > TNT > 2-NT > NB > 3-NT > 2,4-DNT > 2,6-DNT > 4-NT. These results clearly indicate the high selectivity of **TB-Zn-CP** towards phenolic nitroaromatics over other potentially interfering non-phenolic nitroaromatics. We believe that the highly selective binding of phenolic nitroaromatics is ascribed to the strong intermolecular interactions, particular due to the interactions between the –OH group and Lewis basic –N of the Tröger's base structural motif.^5*b*^ The observed differential fluorescence quenching is also indicated by sharp visual colour changes as is evident from the inset in Fig. 5.

**Fig. 6:**
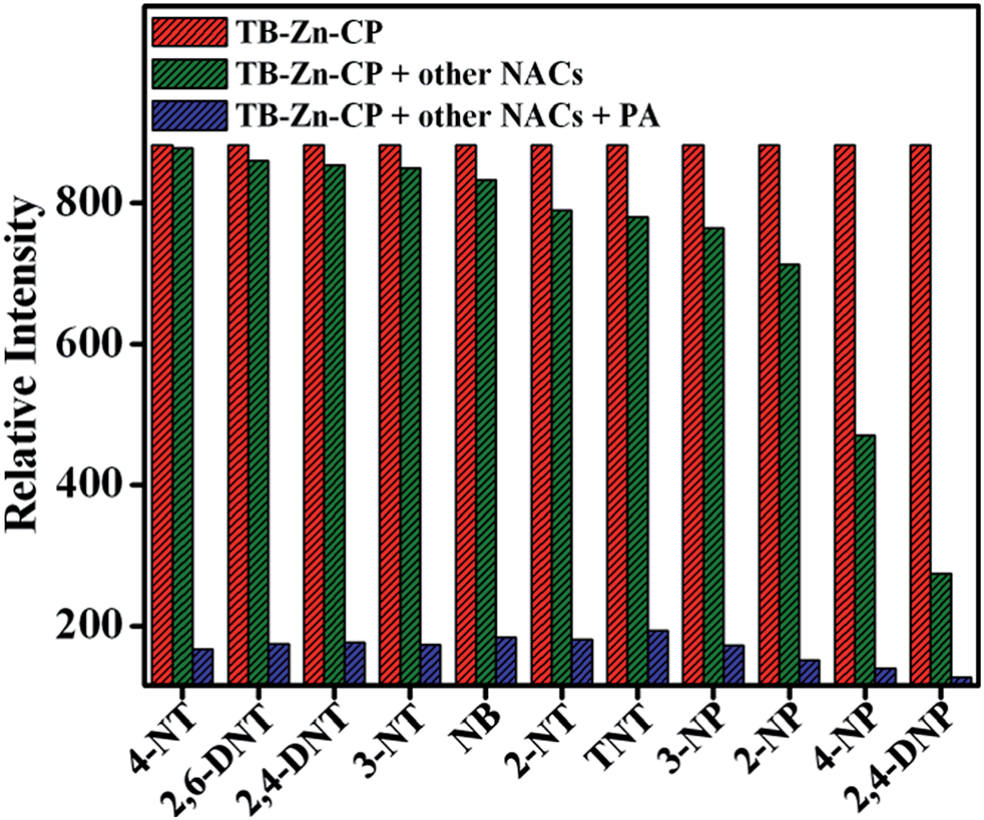
Competitive selective binding affinity of **TB-Zn-CP** towards different NACs in the presence of PA in aqueous medium.

The high selectivity of **TB-Zn-CP** towards these aromatics was further validated by carrying out a competitive fluorescence titration study using PA in the presence of other potentially competing nitroaromatic analytes. The results from this are shown in Fig. 6, where the initial emission (red bar) intensity of **TB-Zn-CP** did not change significantly, except for 4-NP and 2,4-DNP, upon mixing with other competing NACs (green bar). However, the subsequent addition of PA elicited a discernable decrease in the emission intensity (blue bar), which confirmed the discriminative sensing ability of **TB-Zn-CP** towards phenolic nitroaromatics in competing aqueous media. In general, the fluorescence quenching based detection of NACs follow two different mechanisms, *e.g.* static and dynamic or collisional quenching.^9a,36^ The static and dynamic quenching are simple PET or energy transfer from the excited state fluorophores to the analytes; this being characterised by a linear Stern–Volmer plot.^36*a*^ In static quenching, the fluorophore binds with the analyte in the ground state through non-fluorescent charge-transfer complex formation and it solely depends on the extent of binding between the sensor and analyte. Dynamic quenching is an excited state process, in which the analyte binds with the fluorophore in the excited state and it depends on both the rate of collision of and lifetime of the fluorophore. Therefore, the static and dynamic quenching mechanisms can be distinguished by analysing the time-resolved fluorescence decay of the fluorophore at different analyte concentrations and the effect of temperature on the extent of fluorescence quenching.^1b,3b,9a,37^

**Fig. 7:**
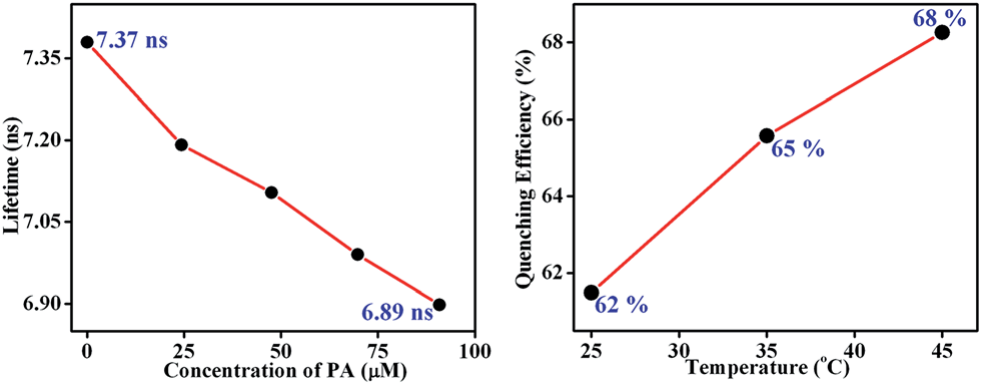
Fluorescence decay profile (left) of the aqueous suspension of **TB-Zn-CP** upon the addition of PA in different concentrations (0–90.9 μM) (left) and extent of fluorescence quenching (right) observed after the addition of PA (47.6 μM) at different temperatures (25–45 °C).

Here, the time-dependent fluorescent decay and temperature dependent fluorescence studies indicated that the observed fluorescence quenching of **TB-Zn-CP**, upon the addition of PA, was due to the dynamic quenching mechanism. As shown in Fig. 7 (left), the decrease in the lifetime (*τ* = 7.37 ns) of **TB-Zn-CP** upon increasing the concentration of PA (0 → 90.9 μM) revealed that PA binds with **TB-Zn-CP** in the excited state. Furthermore, the temperature dependent fluorescence titration study showed that the quenching enhanced as a function of increasing temperature, Fig. 7 (right). The estimated quenching efficiency of **TB-Zn-CP** at 25 °C was *ca.* 62%, being increased to 68% upon elevating the temperature to 45 °C. The increase in quenching efficiency at high temperature is also due to the increased molecular collision between the two species. These two results confirmed that the observed fluorescence quenching occurs through the dynamic quenching mechanistic pathway. The calculated quenching constant varies from 0.40 × 10^11^ M^–1^ S^–1^ to 59.29 × 10^11^ M^–1^ S^–1^ and the results are summarized in Table 1. The higher values of the quenching constant further corroborate the dynamic quenching pathway.^18a,38^

**Table 1:** The quenching constants *K*_q_ and *K*_SV_ of various nitroaromatics for the fluorescence quenching of **TB-Zn-CP**

Analytes	*K*_SV_ (M^–1^) × 10^3^	*K*_q_ (M^–1^ S^–1^) × 10^11^
4-NT	0.30	0.40
2,6-DNT	0.33	0.44
2,4-DNT	0.59	0.80
3-NT	0.47	0.63
NB	1.17	1.58
TNT	1.09	1.47
2-NT	1.53	2.07
3-NP	1.71	2.32
2-NP	5.29	7.17
4-NP	12.6	17.09
2,4-DNP	24.6	33.37
PA	43.7	59.29

### Fluorescence quenching response time

**Fig. 8:**
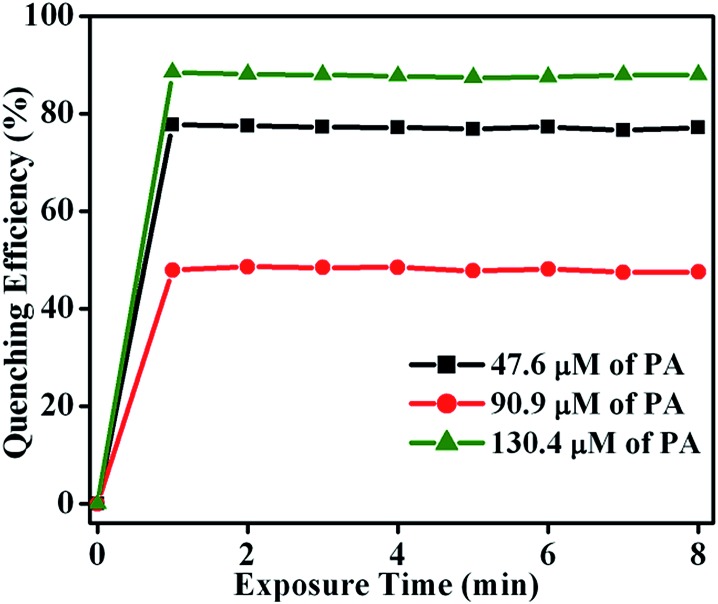
Plot of quenching efficiency as a function of exposure time (0 → 8 min) monitored at different concentrations of PA (47.6 → 130.4 μM).

To determine the fluorescence quenching response time for the detection of NACs by **TB-Zn-CP**, time-dependent fluorescence titration experiments were carried using varyious concentrations of PA (47.6 → 130.4 μM). The plot of quenching efficiency *vs.* exposure time is shown in Fig. 8, demonstrating a fast response time for the sensing of PA. For instance, the addition of PA (130.4 μM) caused ∼90% fluorescence quenching of **TB-Zn-CP** within 60 s of contact time and thus **TB-Zn-CP** can be used as a potential sensor for the quick detection of NACs.

### Sensitivity

**Fig. 9:**
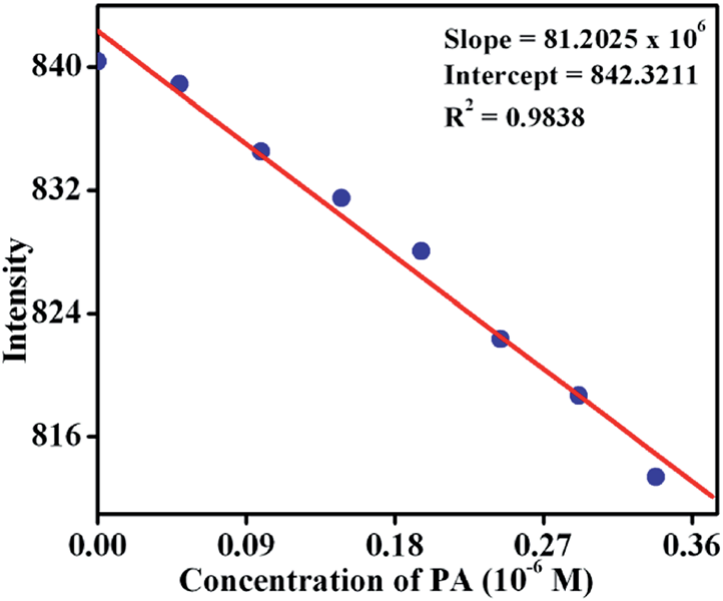
Change in fluorescence intensity of **TB-Zn-CP** at different concentrations of PA.

To establish the sensitivity of **TB-Zn-CP** towards phenolic-nitroaromatics, the fluorescence quenching titration studies were also performed at a very low concentration of PA; the detection limit being estimated by using:Detection limit = 3*σ*/*K*where *σ* is the standard deviation of the initial emission intensity in the absence of the analyte and *K* is the slope of the linear calibration curve. As shown in Fig. 9, the plot of the change in fluorescence intensity *versus* PA concentration, resulted in a linear curve with slope *K* = 81.2 × 10^6^ and a correlation coefficient of *R* ^2^ = 0.9838. The quantitative analysis of the fluorescence titration profile revealed that **TB-Zn-CP** can respond to the presence of PA as low as the 26.3 ppb level of concentration which is comparable with other reported coordination polymer based fluorescence sensors when measured in organic solutions and this level of sensitivity comes under the allowed limit of NACs in drinking water as established by the US EPA.^8,10a^ Hence, the results for **TB-Zn-CP** demonstrate that the material can function both in competitive media and within the recommended limit of detection which is highly significant for practical applications in the field.

### Reversibility

**Fig. 10:**
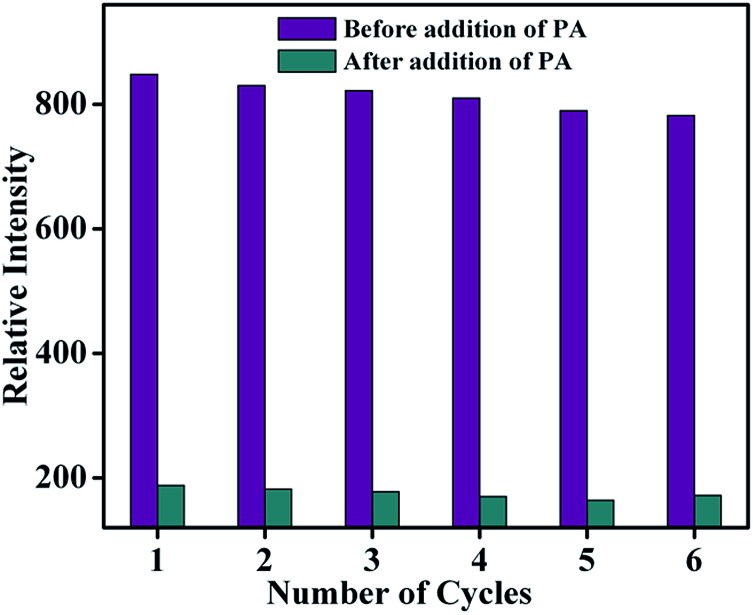
Reproducibility of the quenching ability of **TB-Zn-CP** for PA dispersed in water. The material was recovered by centrifugation after each experiment and washed several times with water.

As far as a real-time application is concerned, the sensing ability of the sensor material must be reversible. The reproducibility of the sensing process of **TB-Zn-CP** towards PA was thus investigated. The emission intensity of **TB-Zn-CP** was first recorded in the presence of PA at 90.9 μM concentration and after each measurement, the material was collected by centrifugation and washed several times with water, or until the centrifugate became colourless. After six cycles of repetition, almost 92% of the initial emission intensity was retained and thus **TB-Zn-CP** showed excellent recyclability as demonstrated in Fig. 10. This study also revealed the excellent photostability of **TB-Zn-CP** demonstrating that the material can be used as a luminescent sensor for a significant number of sensing cycles.^3a,6a,14c,18c^

## Conclusions

Herein we present our results on the design, synthesis and physical evaluation of a nitrogen-rich supramolecular Zn(ii) coordination polymer **TB-Zn-CP** incorporating a 4-amino-1,8-naphthalimide derived Tröger's base structural motif as a sensory material for the detection of nitro-aromatic based explosives. The phase-purity including the morphological features were analysed by using various spectroscopy and microscopy techniques, as well as gas uptake studies. We show that the aqueous suspension of activated **TB-Zn-CP** showed strong fluorescence characteristics, this being assigned to the ICT character of the naphthalimide structure, and this property was used as a selective and discriminative fluorescence sensor for NACs. The gradual addition of NACs caused poor to significant quenching in the fluorescence emission of **TB-Zn-CP**; the observed quenching being ascribed to the excited state energy transfer from **TB-Zn-CP** to the nitroaromatics. The differential fluorescence quenching responses of **TB-Zn-CP** for closely related nitroaromatics, demonstrated significant selectivity of **TB-Zn-CP** for phenolic nitroaromatics.^23*b*^ Notably, the ability of **TB-Zn-CP** to discriminate PA over TNT (both are marginally different in their chemical structure) was an interesting observation that has seldom been seen with other Zn^2+^ based coordination polymers. The time-resolved and temperature dependent fluorescence titration studies confirmed that the fluorescence quenching occurred through dynamic quenching; the detection limit for the sensing of PA being determined to be 26.3 ppb, which is comparable with other d^10^ (Zn^2+^ and Cd^2+^) metal based coordination polymers designed for such applications.^7a,16a^ Recyclability experiments revealed that **TB-Zn-CP** was highly photo-stable and that the sensing was reversible; demonstrating that **TB-Zn-CP** can be used for long-term infield sensing of NACs. In summary, the high selectivity and discriminative sensing ability including superior sensitivity and reversibility make **TB-Zn-CP** a promising sensor material for the rapid detection of phenolic-nitroaromatics.

## Experimental

### Materials

Starting materials were obtained from Sigma Aldrich, Strem Chemicals and Fluka. Solvents used were HPLC grade unless otherwise stated. ^1^H NMR spectra were recorded at 400 MHz using a Bruker Spectrospin DPX-400, with chemical shifts expressed in parts per million (ppm or d) downfield from the standard. ^13^C NMR spectra were recorded at 100 MHz using a Bruker Spectrospin DPX-400 instrument. Infrared spectra were recorded on a Mattson Genesis II FTIR spectrophotometer equipped with a Gateway 2000 4DX2-66 workstation. Mass spectroscopy was carried out using HPLC grade solvents. Mass spectra were determined by detection using Electrospray on a Micromass LCT spectrometer, using a Water's 9360 to pump solvent. The system was controlled by Mass Lynx 3.5 on a Compaq Deskpro workstation.

### Synthesis

#### *N*-(5-Dimethyl-isophthalate)-4-nitro-1,8-naphthalimide (**1**)

4-Nitro-1,8-naphthalic anhydride (3.50 g, 14.4 mmol, 1.0 eq.), dimethyl-5-aminoisophthalate (6.02 g, 28.8 mmol, 2.0 eq.) and imidazole (21.57 g, 316.8 mmol, 22.0 eq.) were dissolved in chloroform (150 mL) and stirred at reflux for 2 h at 80 °C. The solvents were removed under reduced pressure and the residue was suspended into ice-cold ethanol and sonicated for 15 min. The solution was filtered and the product was washed with ice cold ethanol (3 × 150 mL), dried *via* vacuum filtration and isolated as an off-white powder (4.9 g, 11.3 mmol, 78%) after triturating with CHCl_3_. Melting point 326–327 °C (decomp.). HRMS (*m*/*z*): calcd for C_22_H_14_N_2_O_8_ 434.0828; found 434.2694 [M – H]^–^. ^1^H NMR (600 MHz, (CD_3_)_2_SO) *δ* 8.80 (1H, d, *J* = 8.3 Hz, Ar-H), 8.67 (1H, d, *J* = 7.6 Hz, Ar-H), 8.65 (1H, d, *J* = 7.8 Hz, Ar-H), 8.62 (1H, d, *J* = 7.8 Hz, Ar-H), 8.60 (1H, t, *J* = 6.6 Hz, Ar-H), 8.38 (2H, d, *J* = 1.54 Hz, Ar-H), 8.12 (1H, dd, *J* = 10.8 Hz, 8.3 Hz, Ar-H), 3.92 (6H, s, CH_3_–H); ^13^C NMR (151 MHz, (CD_3_)_2_SO) *δ* 170.1, 164.1, 163.2, 162.4, 161.2, 149.3, 136.7, 134.5, 131.6, 131.1, 130.1, 129.4, 128.9, 128.8, 127.2, 124.2, 123.4, 52.6. IR *ν*_max_ (neat sample, cm^–1^) 3106, 3081, 2957, 1729, 1709, 1669, 1623, 1582, 1526, 1432, 1353, 1334, 1242, 1196, 1156, 1132, 988, 908, 882, 843, 784, 751, 682. UV-vis (DMSO) *λ*_max_(*ε*) [nm (M^–1^ cm^–1^)]: 350 (3953). Non-emissive due to the lack of internal charge transfer (ICT) transition.

#### *N*-(5-Dimethyl-isophthalate)-4-amino-1,8-naphthalimide (**2**)

Compound **1** (200 mg, 0.460 mmol, 1 eq.) was reduced by catalytic hydrogenation using Pd/C (10%, 20 mg) at 3 atm of H_2_ in C_2_H_5_OH : DMF (9 : 1, 10 mL) for 12 h. The mixture was diluted with DCM : CH_3_OH (1 : 1, 400 mL) and stirred in the dark for 1 h. The reaction mixture was then filtered through celite and washed several times with DCM : CH_3_OH (1 : 1) until the washings ran clear. The solvents were removed under reduced pressure to isolate compound **2** (48 mg, 0.119 mmol, 25%) as an orange solid after trituration with cold-diethylether. Melting point 317–319 °C (decomp.). HRMS (*m*/*z*): calcd for C_22_H_15_N_2_O_6_ 403.1008; found 403.0932 (M – H)^–^; ^1^H NMR (600 MHz, (CD_3_)_2_SO) *δ* 8.64 (1H, d, *J* = 8.11 Hz, Ar-H), 8.53 (1H, s, Ar-H), 8.44 (1H, d, *J* = 7.70 Hz, Ar-H), 8.21 (2H, d, *J* = 7.50 Hz, Ar-H), 8.19 (1H, s, Ar-H), 7.69 (1H, t, Ar-H), 7.53 (2H, s, NH_2_), 6.89 (1H, d, *J* = 8.20 Hz, Ar-H), 3.91 (6H, s, CH_3_–H); ^13^C NMR (151 MHz, (CD_3_)_2_SO) *δ* 165.9, 164.9, 164.0, 154.0, 138.7, 135.7, 135.5, 135.1, 132.1, 131.8, 131.3, 130.6, 129.9, 124.9, 123.2, 120.5, 109.1, 108.6, 53.6. IR *ν*_max_ (neat sample, cm^–1^) 3427, 3368, 3266, 1732, 1688, 1639, 1580, 1532, 1428, 1373, 1329, 1248, 1145, 996, 894, 835, 755, 685. UV-vis (DMSO) *λ*_max_(*ε*) [nm (M^–1^ cm^–1^)]: 273 (11 423), 438 (7538). Emission (DMSO) *λ*_max_ (nm) = 533.

#### Bis-[*N*-(5-dimethyl-isophthalate)]-9,18-methano-1,8-naphthalimide-[*b*,*f*][1,5]diazocine (**3**)

Compound **2** (150 mg, 0.371 mmol, 1 eq.) and paraformaldehyde (17 mg, 0.556 mmol, 1.5 eq.) were flushed with argon. Trifluoroacetic acid (3 mL) was added at 0 °C and the solution was stirred at room temperature for overnight under an inert atmosphere. The reaction mixture was added dropwise to aqueous ammonium hydroxide (100 mL) at 0 °C and ammonia solution was added until a pH > 10 was achieved. DCM (200 mL) was added and the organic solution was extracted and washed with saturated NaHCO_3_, brine, and H_2_O. The solution was dried over MgSO_4_ and the solvents were removed under reduced pressure. The solid was triturated in Et_2_O and isolated *via* filtration as an orange solid (64 mg, 0.076 mmol, 20%). Melting point 293–295 °C (decomp.). HRMS (*m*/*z*): calcd for C_47_H_33_N_4_O_12_ 845.2017; found 845.2095 (M + H)^+^; ^1^H NMR (600 MHz, (CD_3_)_2_SO) *δ* 8.77 (2H, d, *J* = 7.90 Hz, Ar-H), 8.49 (2H, t, *J* = 6.00 Hz, Ar-H), 8.46 (2H, d, *J* = 7.40 Hz, Ar-H), 8.19 (4H, s, Ar-H), 8.12 (2H, s, Ar-H), 7.97 (2H, t, *J* = 12.00 Hz, Ar-H), 5.24 (2H, d, *J* = 17.57 Hz, CH_2_N–H), 4.79 (2H, s, NCH_2_N–H), 4.74 (2H, d, *J* = 17.47 Hz, CH_2_N–H), 3.89 (12H, s, CH_3_–H); ^13^C NMR (151 MHz, (CD_3_)_2_SO) *δ* 164.8, 163.6, 163.0, 149.2, 137.1, 134.6, 130.9, 130.5, 130.2, 129.2, 128.1, 127.1, 126.7, 126.1, 123.1, 118.2, 52.6; IR *ν*_max_ (neat sample, cm^–1^) 2953, 1707, 1667, 1596, 1347, 1239, 1180, 998, 783, 753. UV-vis (DMSO) *λ*_max_(*ε*) [nm (M^–1^ cm^–1^)]: 347 (13 058), 390 (15 503). Emission (DMSO) *λ*_max_ (nm) = 545.

#### Bis-[*N*-(1,3-benzenedicarboxylic acid)]-9,18-methano-1,8-naphthalimide-[*b*,*f*][1,5]diazocine (**L**)

Compound **3** (50 mg, 0.059 mmol, 1 eq.), was dissolved in THF : MeOH (4 : 1, 20 mL) and stirred for 10 min. KOH (aq.) (33 mg, 0.592 mmol, 10 mL, 10 eq.) was added and the solution was refluxed at 60 °C for 12 h. Solvents removed under reduced pressure, leaving water. The solution was acidified using HCl (1 M) until pH = 1 as achieved. The acidic solution was left to stir for 12 h and was filtered, washed with water, dried with diethyl ether and isolated as a yellow powder (30 mg, 0.038 mmol, 65%). Melting point 262–263 °C (decomp.). HRMS (*m*/*z*): calcd for C_43_H_23_N_4_O_12_ 787.1319; found 787.1308 (M – H)^–^; ^1^H NMR (600 MHz, (CD_3_)_2_SO) *δ* 13.41 (4H, broad, COOH), 8.81 (2H, d, *J* = 8.51 Hz, Ar-H), 8.51 (2H, m, Ar-H), 8.47 (2H, s, Ar-H), 8.15 (2H, s, *J* = 10.00 Hz, Ar-H), 8.11 (4H, s, Ar-H), 7.97 (2H, t, *J* = 12.00 Hz, Ar-H), 5.24 (2H, d, *J* = 17.58 Hz, NCH_2_–H), 4.78 (2H, s, NCH_2_N), 4.74 (2H, d, *J* = 17.58 Hz, NCH_2_–H); ^13^C NMR (151 MHz, (CD_3_)_2_SO) *δ* 166.9, 164.6, 164.0, 150.1, 137.6, 135.2, 133.0, 131.5, 131.1, 130.5, 130.2, 129.0, 128.0, 127.7, 127.0, 124.0, 119.2, 67.0, 57.8. IR *ν*_max_ (neat sample, cm^–1^) 3350, 2971, 1705, 1659, 1596, 1459, 1373, 1241, 1191, 1126, 948, 814, 785, 678. UV-vis (DMSO) *λ*_max_(*ε*) [nm (M^–1^ cm^–1^)]: 347 (13 939), 392 (16 650). Emission (DMSO) *λ*_max_ (nm) = 532.

#### Synthesis of **TB-Zn-CP**

A DMF (2 mL) solution of Zn(OAc)_2_·2H_2_O (16.7 mg, 0.08 mmol, 1.0 eq.) was added drop-wise to a clear DMF (2 mL) solution of **L** (30.0 mg, 0.04 mmol, 0.5 eq.) with continues stirring. Upon addition of Zn(OAc)_2_·2H_2_O, the clear orange solution of **L** gradually turned turbid, indicating the formation of coordination polymers, and the turbid solution was stirred gently for further 6 hours at room temperature to complete the reaction. The orange colloids formed were centrifuged, thoroughly washed with DMF (3 × 5 mL) to remove the unreacted starting materials, and then dried under vacuum for 6 h. The isolated orange colloidal particles were characterized by different techniques. Isolated yield = 28 mg (73%). Elemental analysis calculated for C_43_H_20_N_4_O_12_Zn·2H_2_O·DMF: C, 57.60; H, 3.26; N, 7.30; found: C, 57.54; H, 3.73; N, 7.33. FTIR *ν*_max_ (neat sample, cm^–1^) 3434, 2981, 2157, 1657, 1573, 1458, 1402, 1371, 1249, 923, 785, 678. UV-vis (H_2_O) *λ*_max_(*ε*) [nm (M^–1^ cm^–1^)]: 351 (7485), 380 (8374). Emission (H_2_O) *λ*_max_ (nm) = 520.
