# Supplementary material for: A supramolecular Tröger's base derived coordination zinc polymer for fluorescent sensing of phenolic-nitroaromatic explosives in water
Source: Chem Sci. 2016 Oct 25;8(2):1535–46. doi: 10.1039/c6sc04367d (PMC5452275; doi:10.1039/c6sc04367d)
Supplement: Supplementary file 1 [file SC-008-C6SC04367D-s001.pdf]

Electronic Supplementary Material (ESI) for Chemical Science.
This journal is © The Royal Society of Chemistry 2016

ESI

Supramolecular Tröger's base derived coordination zinc polymer for fluorescent sensing of phenolic-nitroaromatics explosives in water

Sankarasekaran Shanmugaraju,[a]* Charlyne Dabadie,[a] Kevin Byrne,[b] Aramballi J. Savyasachi,[a] Deivasigamani Umadevi,[a] Wolfgang Schmitt,[b] Jonathan A. Kitchen[c] and Thorfinnur Gunnlaugsson[a]*

Experimental section

Materials: All solvents and chemicals were purchased from various commercial sources and used without further purification. 4-Nitro-1,8-naphthalic anhydride, Dimethyl 5-aminoisophthalate, Imidazole, Palladium on carbon (10 wt% loading), Paraformaldehyde, Trifluoroacetic acid, Potassium hydroxide and all nitroaromatic analytes were purchased from Sigma-Aldrich and used as received. Deuterated solvents $(CD_3)_2SO$, $CDCl_3$, CD_2Cl_2) used for NMR analysis were purchased from Apollo Scientific or Sigma-Aldrich.

*(**Caution!** Nitroaromatic analytes are classified as secondary chemical explosives and should be handled only in small quantity)*

Methods: Melting point was determined using an Electrochemical IA9000 digital melting point apparatus. The elemental analysis for C, H and N was performed on an Exeter analytical CE-450 elemental analyzer in University College Dublin. Infrared spectra were recorded on a Perkin Elmer spectrum on FT-IR spectrometer equipped with a universal ATR sampling accessory. The solution phase ^1H and ^{13}C NMR spectrum was recorded at 400 MHz using an Agilant Technologies 400-MR NMR spectrometer. Chemical shifts are reported in ppm with the deuterated solvents as the internal reference. All NMR spectra were carried out at 293 K. Mass-spectrometry was carried out using HPLC grade solvents. Electrospray mass spectra were determined on a Micromass LCT spectrometer and high-resolution mass spectra were

determined relative to a standard of leucine enkephaline. Maldi-Q-TOF mass spectra were carried out on a MALDI-Q-TOF-premier and high-resolution mass spectrometry was performed using Glu-Fib with an internal reference peak of *m/z* 1570.6774. Thermal gravimetric analysis was performed on Perkin Elmer Pyrus 1 TGA equipped with an ultra-microbalance with sensitivity of 0.1 μg. The temperature range is from 25ºC to 800ºC with a scan rate of 10ºC/min under N_2 purge. Powder X-ray diffraction analysis was performed using a Miniflex II Rigaku diffractometer with Ni-filtered Cu Kα radiation (λ = 1.54 Å). The tube voltage and tube current used were 30 Kv and 15 mA, respectively. Each sample was scanned over a 2θ range of 5°–40° with a step size of 0.05°/s. Morphology and particle size of polymerized material was imaged by field emission scanning electron microscopy (FE-SEM) using Zeiss ULTRA Plus with an SE2 or in-lens detector and atomic force microscopy (AFM) in the Advanced Microscopy Laboratory, CRANN, Trinity College Dublin. The samples for FE-SEM/AFM were prepared by drop-casting the aqueous suspension (1 mg in 100 μL of millipore water) of as-synthesized **TB-Zn-CP** on silica wafers, then coated with Au and dried under vacuum before the imaging. UV-visible absorption spectra were recorded in 1 cm quartz cuvettes (Hellma) on a Varian Cary 50 spectrometer. Baseline correction was applied for all spectra. Emission spectra were recorded on a Varian Cary Eclipse Fluorimeter. The temperature was kept constant throughout the measurements at 298 K by using thermostated unit block.

Gas uptake measurements: All the gas (N_2, H_2 and CO_2) adsorption measurements were carried out on a Quantachrome AUTOSORP-IQ automated gas sorption analyzer. The as-synthesized sample was immersed in diethylether for 3 days. During the immersion, the diethyl ether was refreshed 3 times with fresh diethyl ether and the resulting diethylether exchanged polymer was

transferred to a quartz cell. The sample was evacuated under vacuum at 30°C for 3h and then slowly heated to 100°C over 10 h and held at 100°C for a further 5 h. Ultrahigh-purity grade N_2, H_2 and CO_2 were used in all adsorption measurements. The isotherms were measured in a water-ice-acetone bath to reach 273K. The surface area of **TB-Zn-CP** was calculated by *Brunauer-Emmett-Teller* (BET) method and the pore size distribution was calculated using the NL-DFT model.

Preparation of the aqueous suspension of TB-Zn-CP: 2.5 mg was placed in a 10 mL standard measuring flask and 10 mL millipore water was added. Then, the mixture was sonicated for 30 minutes and aged for 3 days to get uniform suspension for the fluorescence titrations.

Fluorescence titration experiments: 200 µL of the suspension of **TB-Zn-CP** in water was taken in quartz cuvette and 1800 µL of water was added to it. During the fluorescence titration, 1 mM solution of different nitroaromatic explosives was added (0.0 µM – 90.9 µM) in an incremental fashion (20 µL each time) to a 2 mL aqueous suspension of **TB-Zn-CP**. The emission intensity was monitored after each addition. For all the fluorescence titration experiments, the excitation wavelength was 380 nm and the emission spectra were recorded in the range of 390-750 nm. The percentage of quenching efficiency was calculated from the following equation:

$$\text{Quenching efficiency (\%)} = (I_0\text{-}1)/I_0 \times 100$$

Where, I_0 is the initial emission intensity of the suspension of **TB-Zn-CP** in water and I is the intensity after addition of the analyte.

Characterization of Trögers base Linker (L) and it's Precursors:

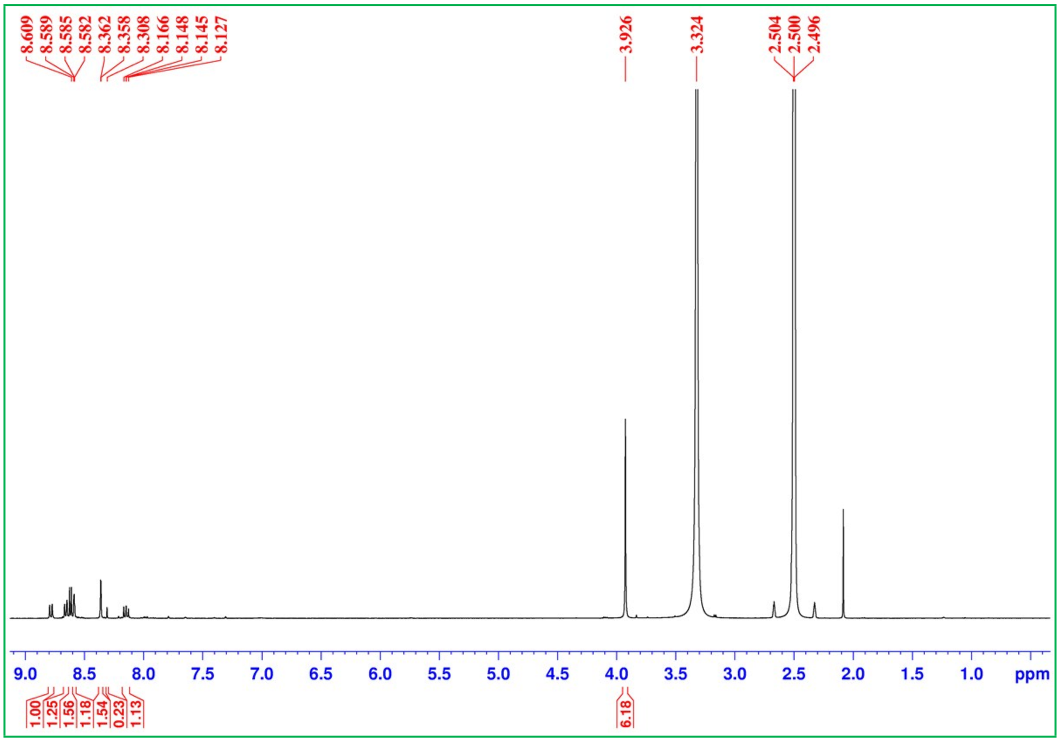

Figure S1. ^{1}H NMR spectrum of **1** (600 MHz, $(CD_3)_2SO$).

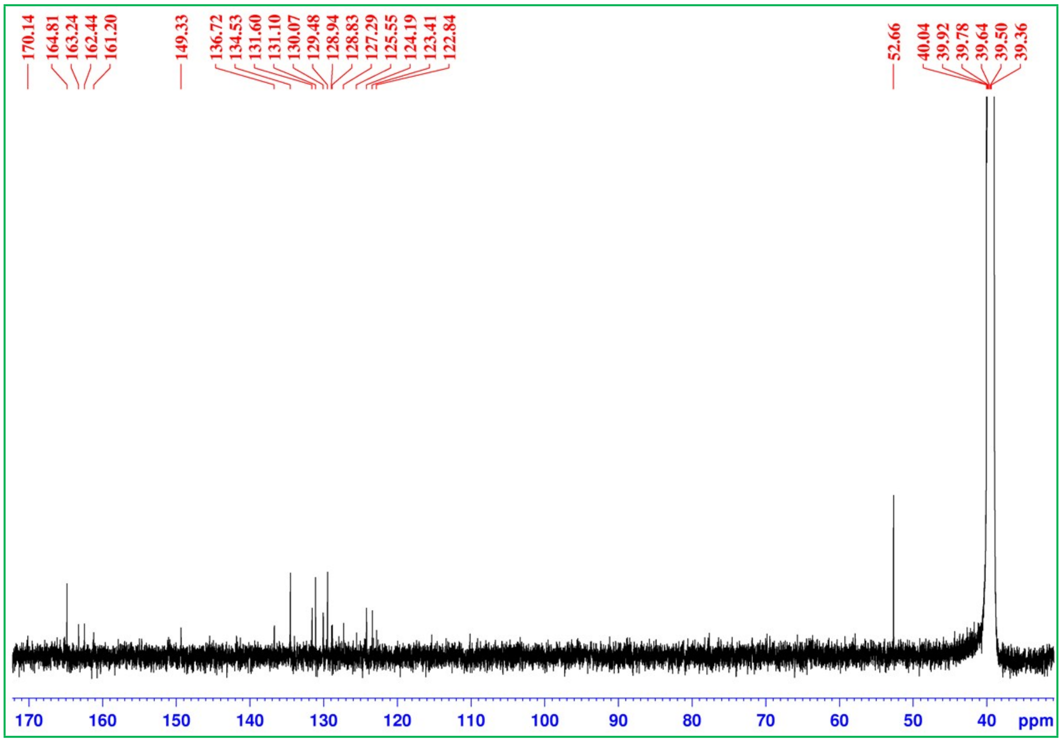

Figure S2. ^{13}C NMR spectrum of **1** (151 MHz, $(CD_3)_2SO$).

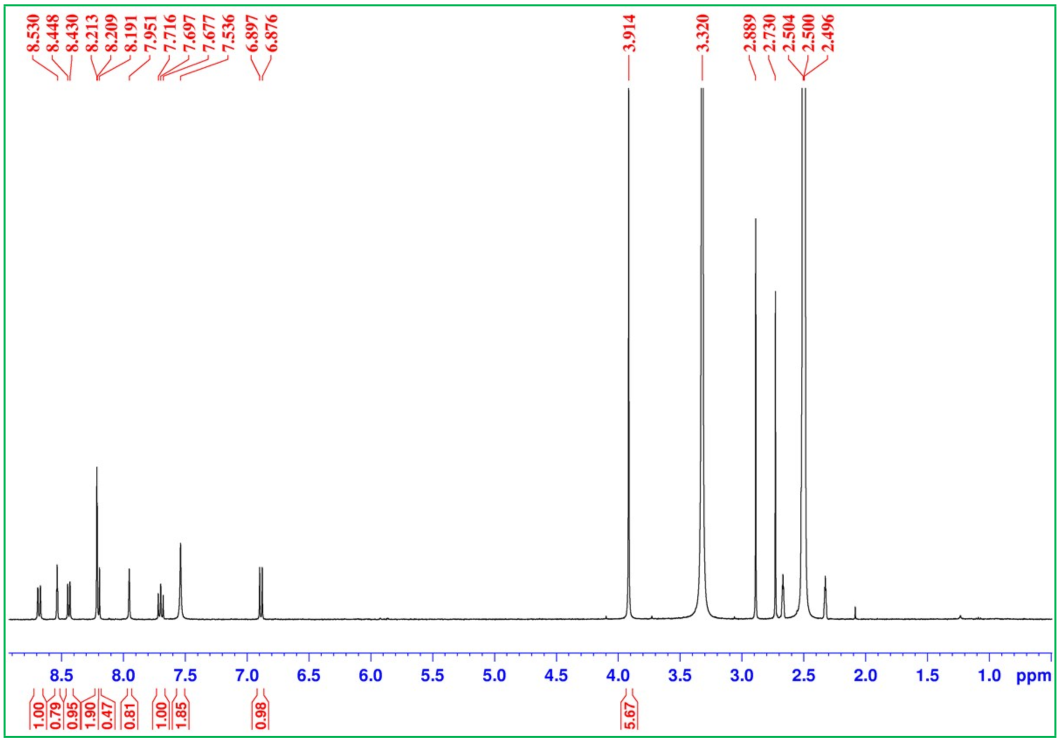

Figure S3. ^{1}H NMR spectrum of **2** (600 MHz, $(CD_3)_2SO$).

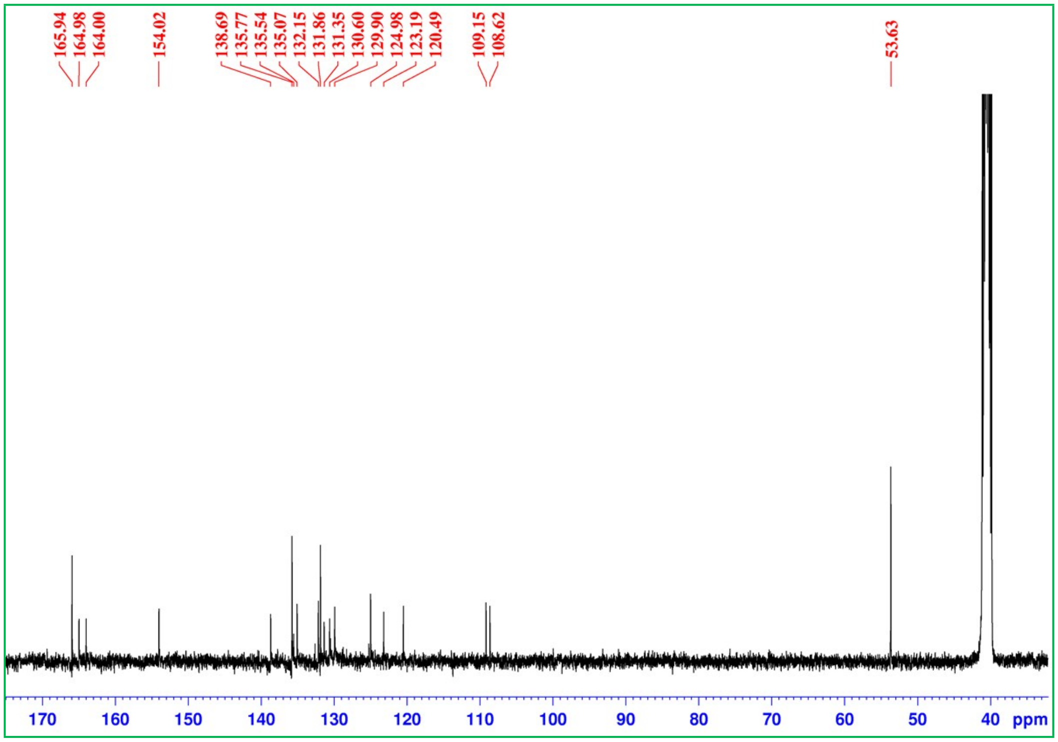

Figure S4. ^{13}C NMR spectrum of **2** (151 MHz, $(CD_3)_2SO$).

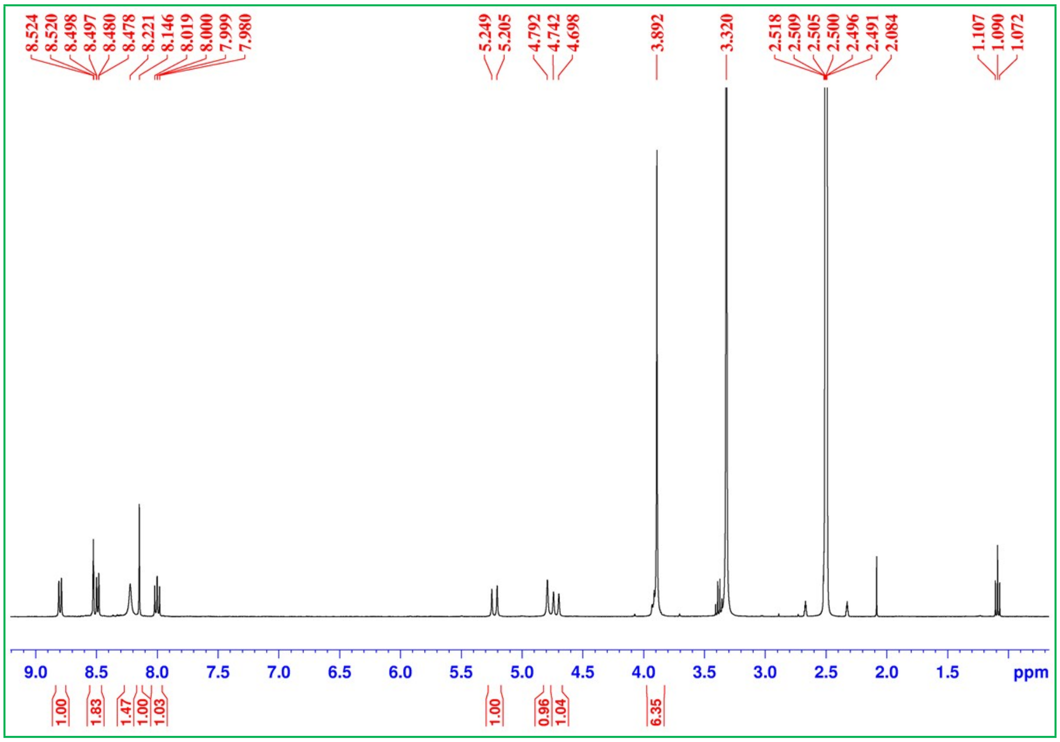

Figure S5. ^{1}H NMR spectrum of **3** (600 MHz, $(CD_3)_2SO$).

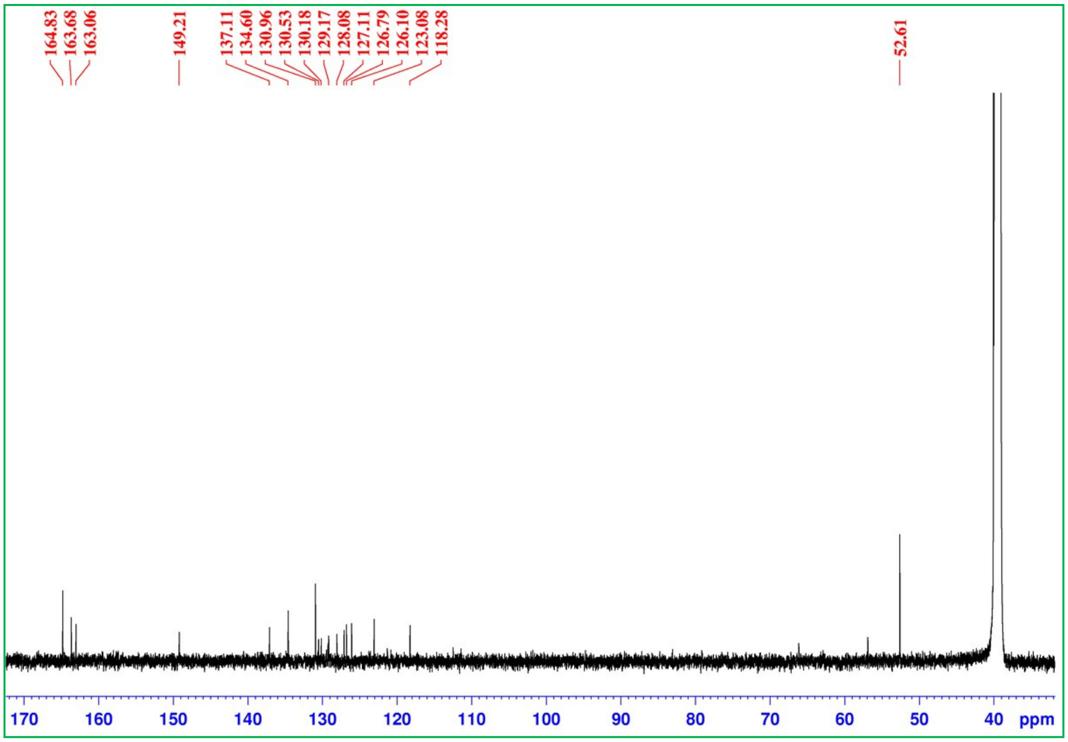

Figure S6. ^{13}C NMR spectrum of **3** (151 MHz, $(CD_3)_2SO$).

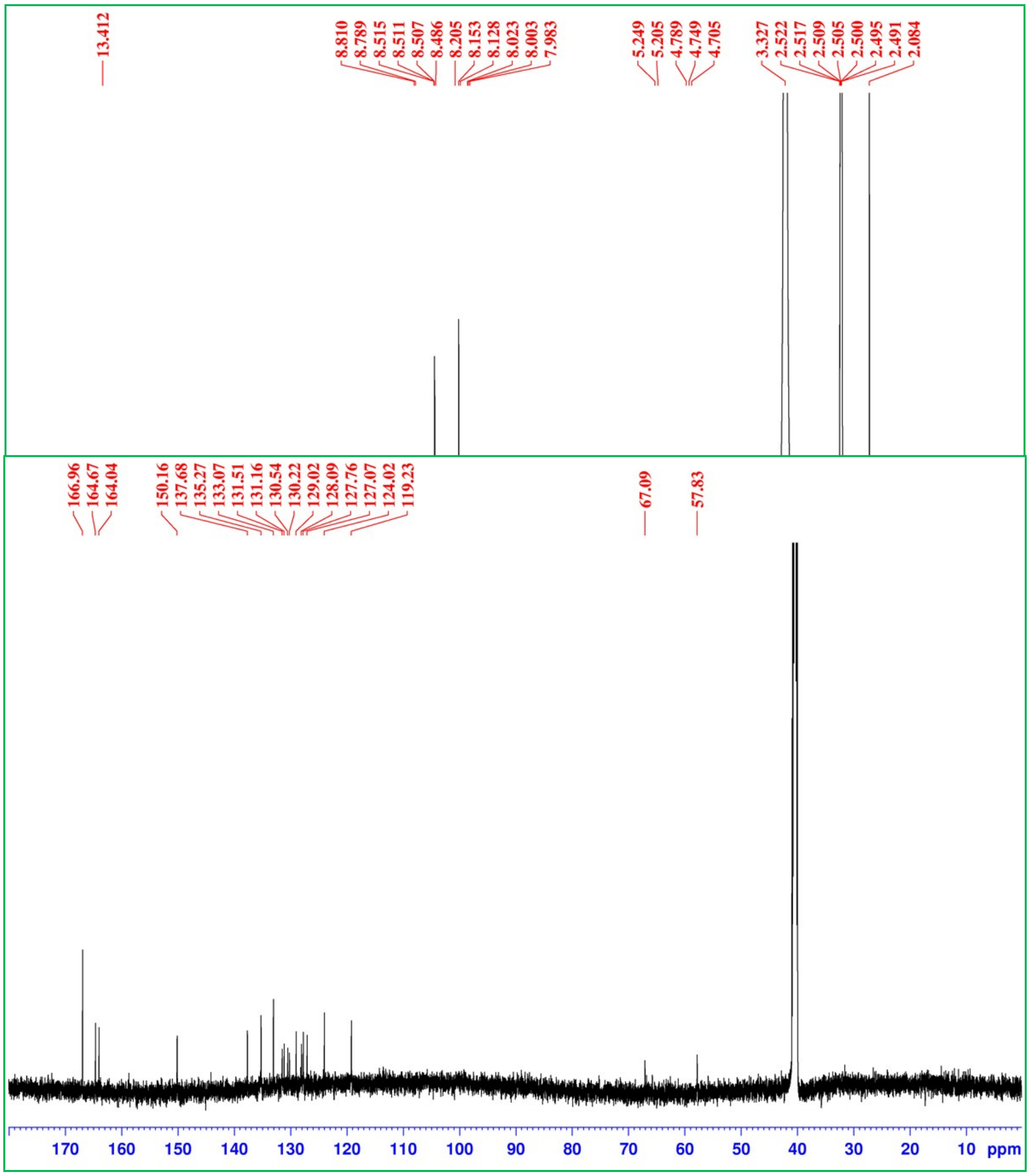

Figure S8. ^{13}C NMR spectrum of **L** (151 MHz, $(CD_3)_2SO$).

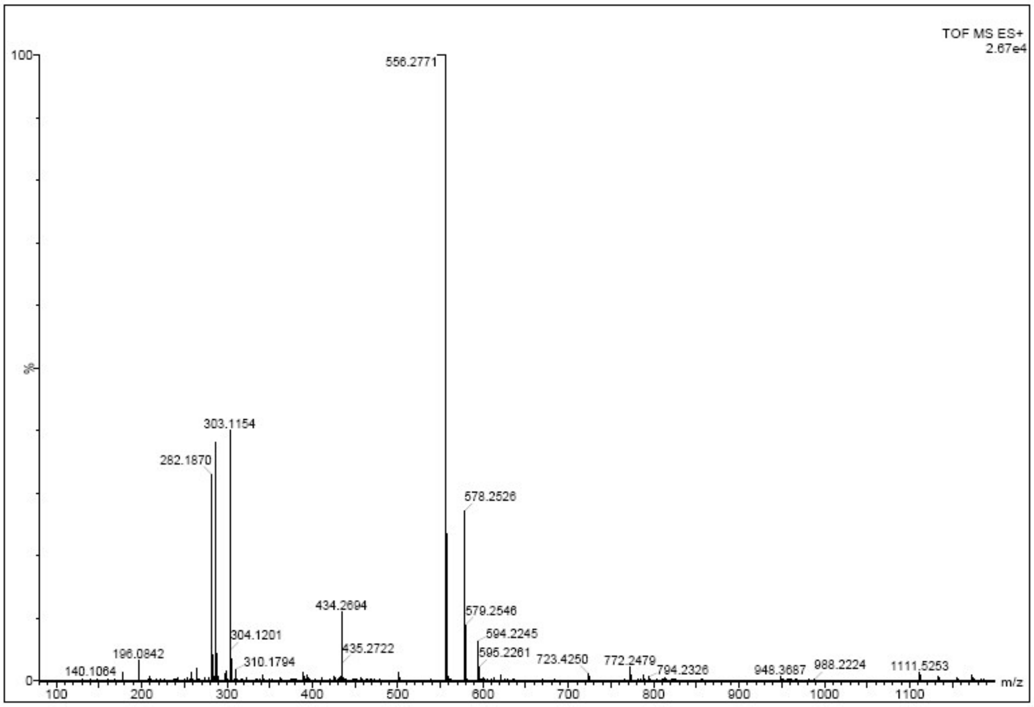

Figure S9. HRMS spectrum of **1** recorded in DMSO.

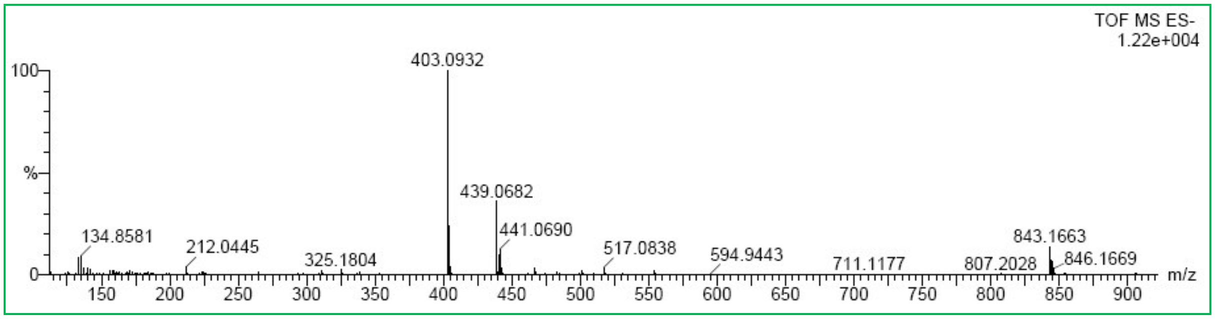

Figure S10. HRMS spectrum of **2** recorded in DMSO.

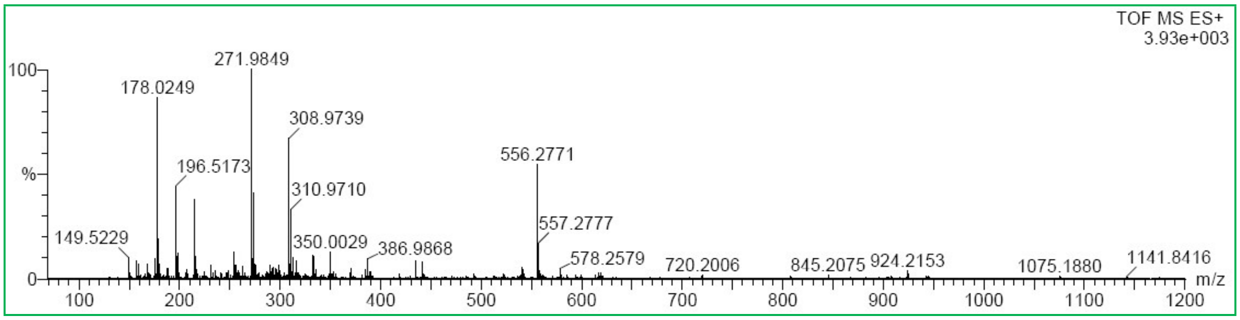

Figure S11. HRMS spectrum of **3** recorded in DMSO.

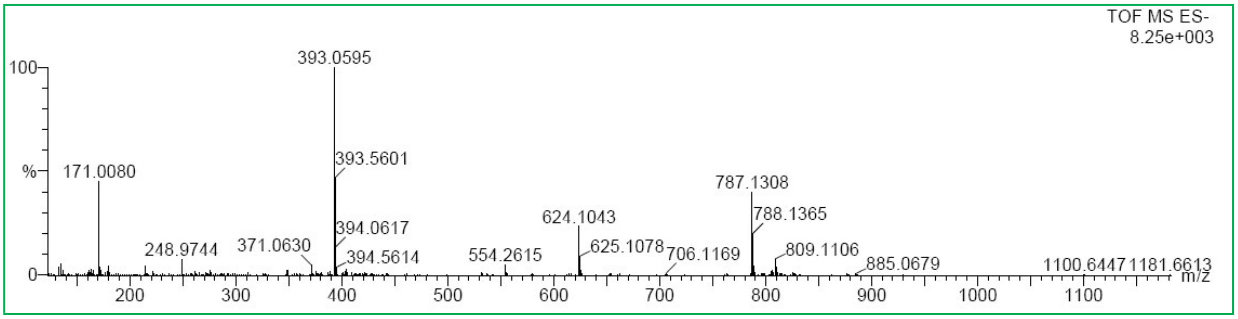

Figure S12. HRMS spectrum of **L** recorded in DMSO.

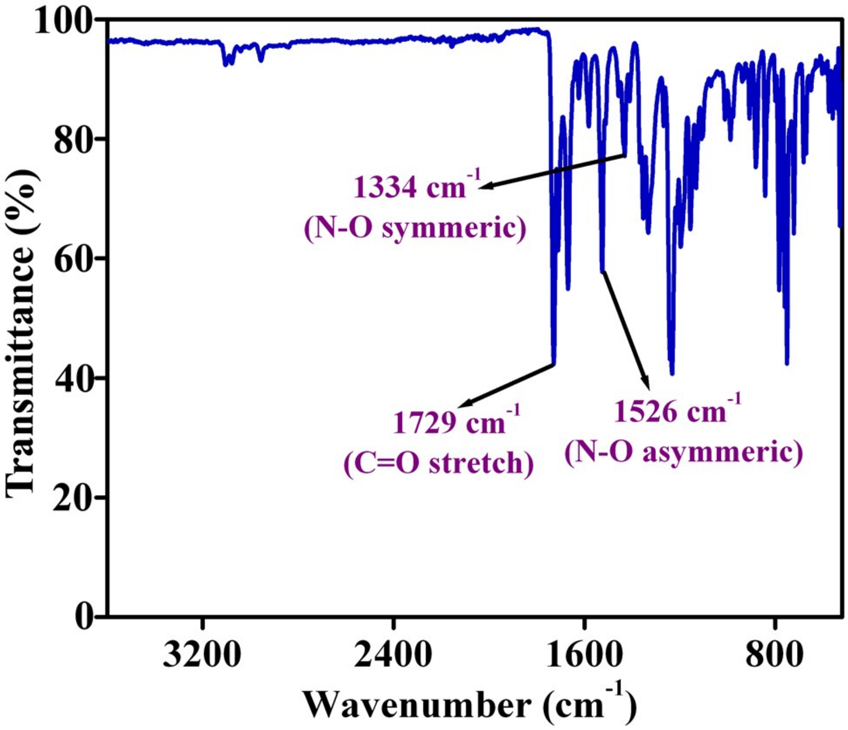

Figure S13. Infrared spectrum of **1**.

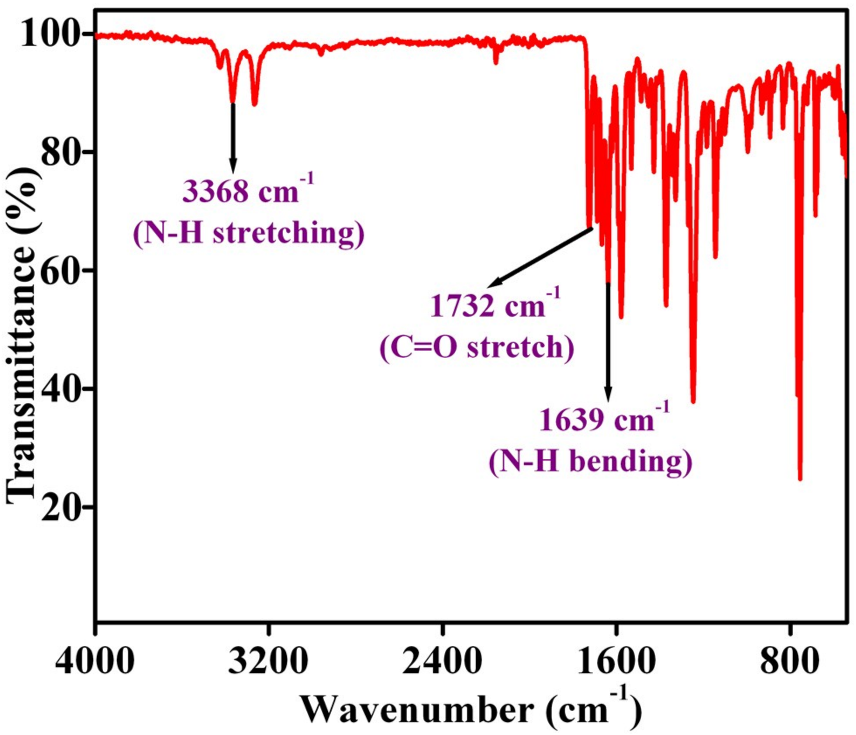

Figure S14. Infrared spectrum of **2**.

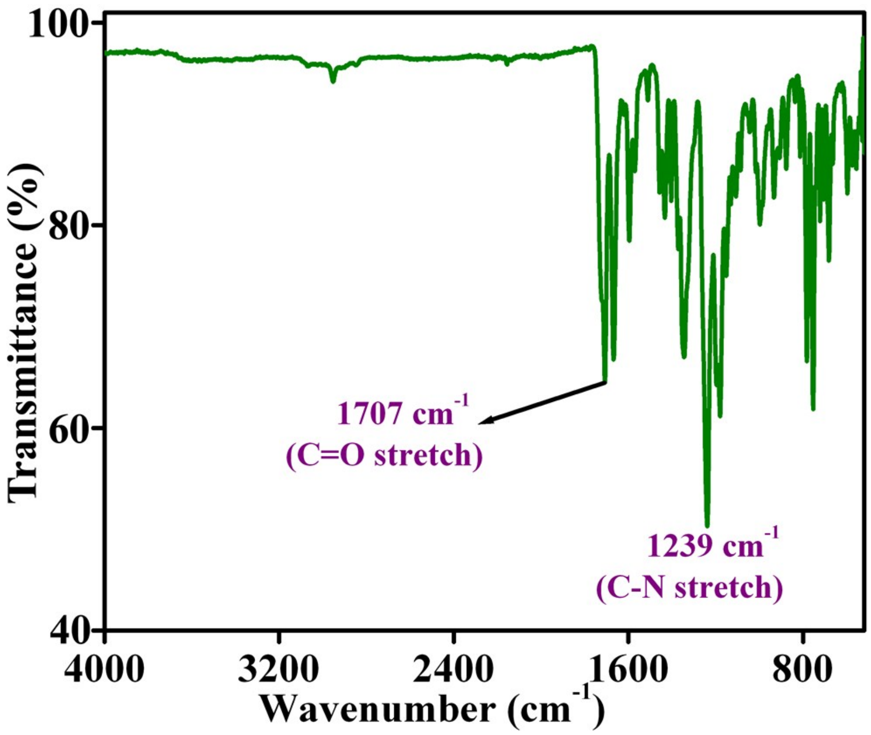

Figure S15. Infrared spectrum of **3**.

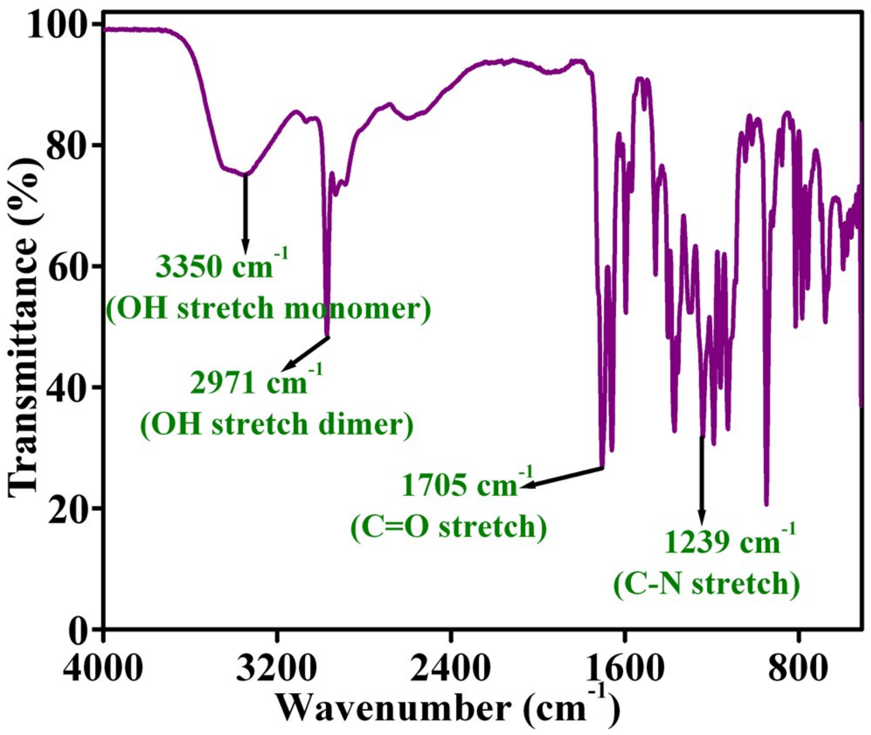

Figure S16. Infrared spectrum of **L**.

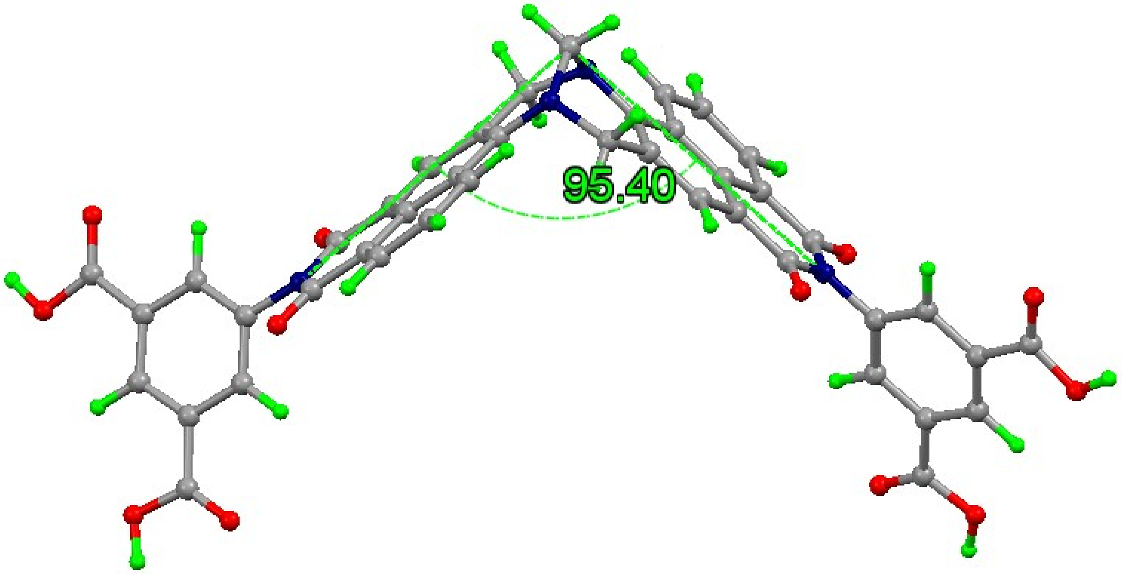

Figure S17. Energy minimized structure of V-Shaped Trögers base linker **L** (grey = C, red = O, blue = N, green = H).

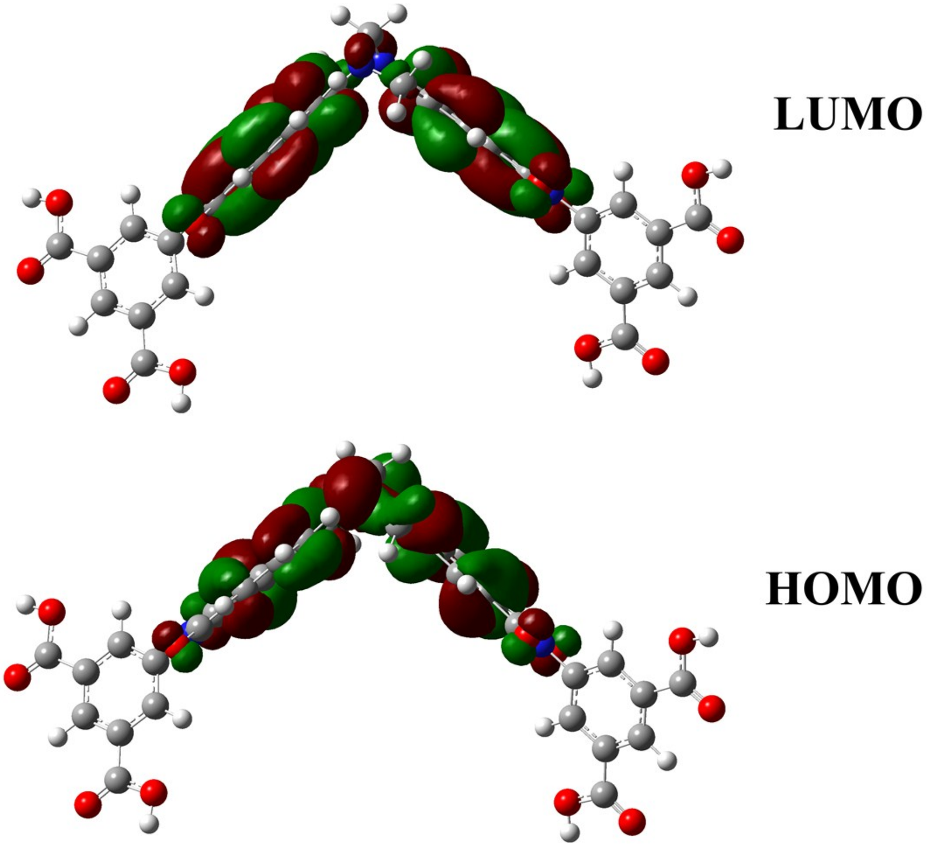

Figure S18. Frontiers molecular orbitals of **L** generated from the energy minimized structure.

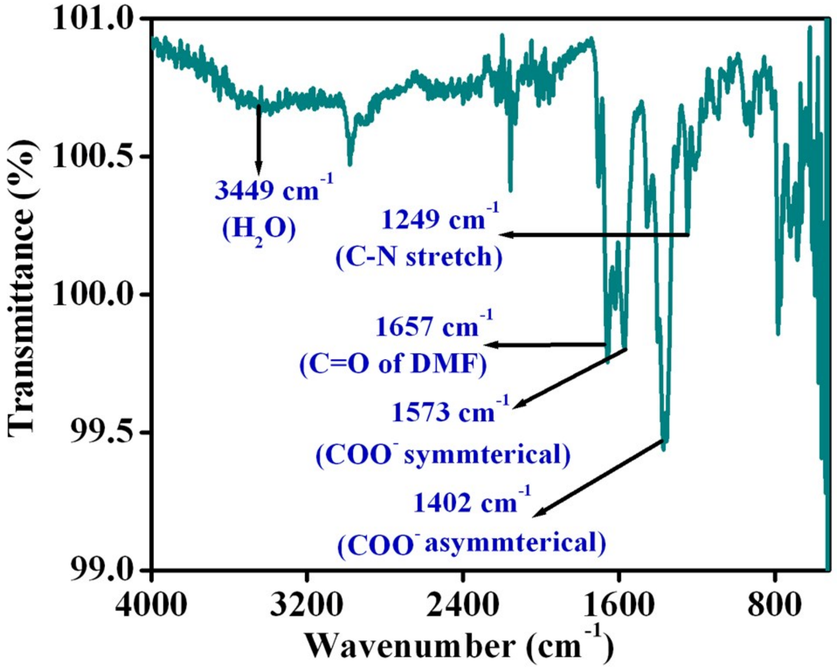

Figure S19. Infrared spectrum of **TB-Zn-CP**.

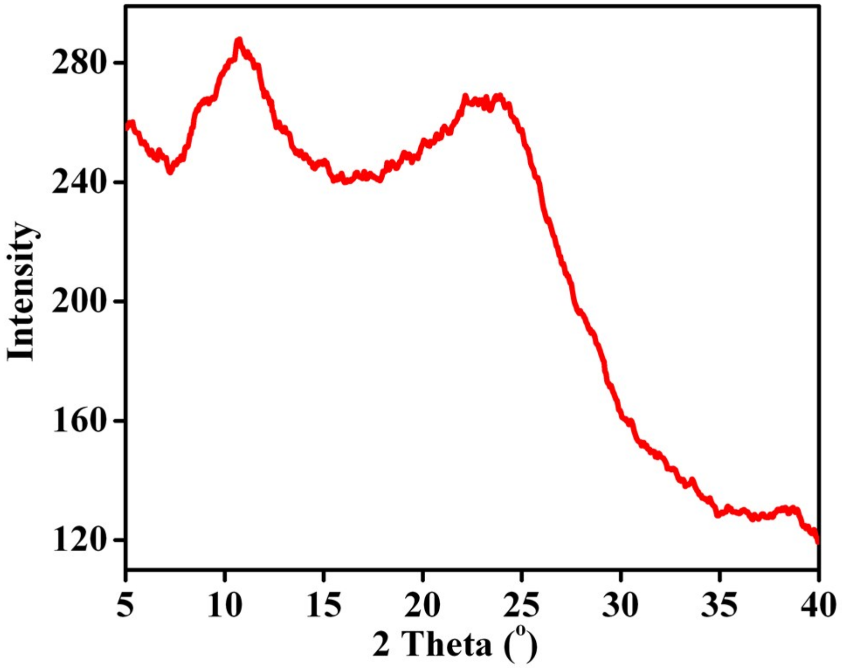

Figure S20. Powder diffraction pattern of as synthesized **TB-Zn-CP**.

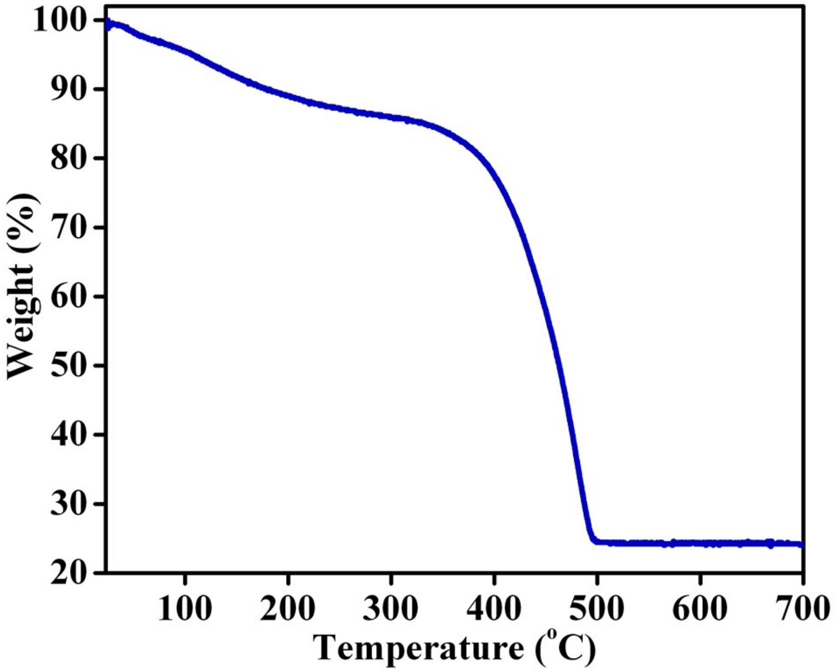

Figure S21. TGA analysis of **TB-Zn-CP** with a heating rate of 5ºC/min.

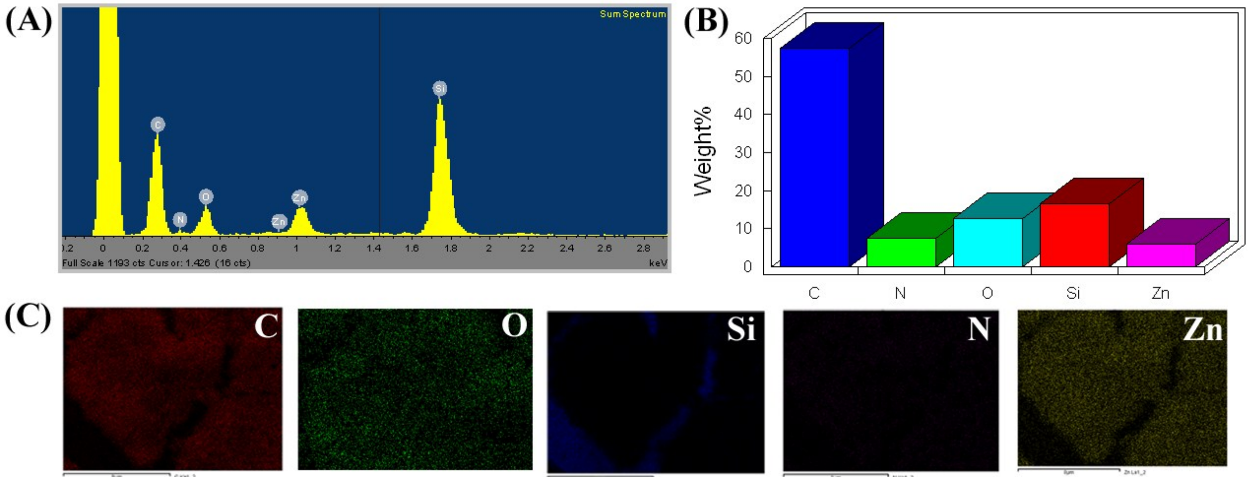

Figure S22. (A) Energy-dispersive X-ray (EDX) data of **TB-Zn-CP** and the quantitative results (B). Quanta mapping images (C) of each elements presence in **TB-Zn-CP.**

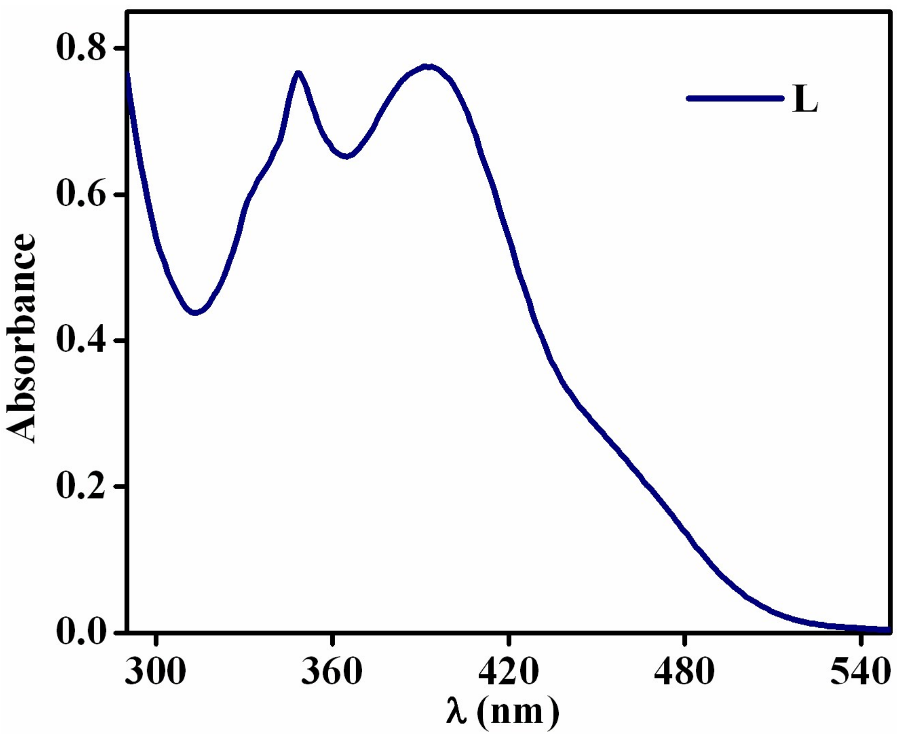

Figure S23. UV-visible absorption spectrum of **L** dispersed in water.

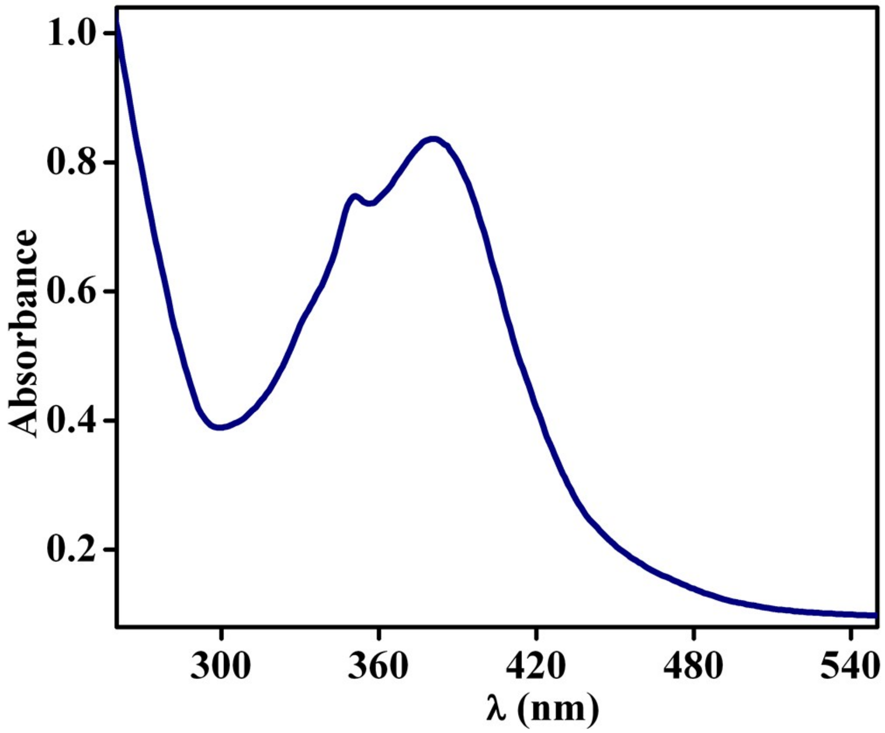

Figure S24. UV-visible absorption spectrum of **TB-Zn-CP** dispersed in water.

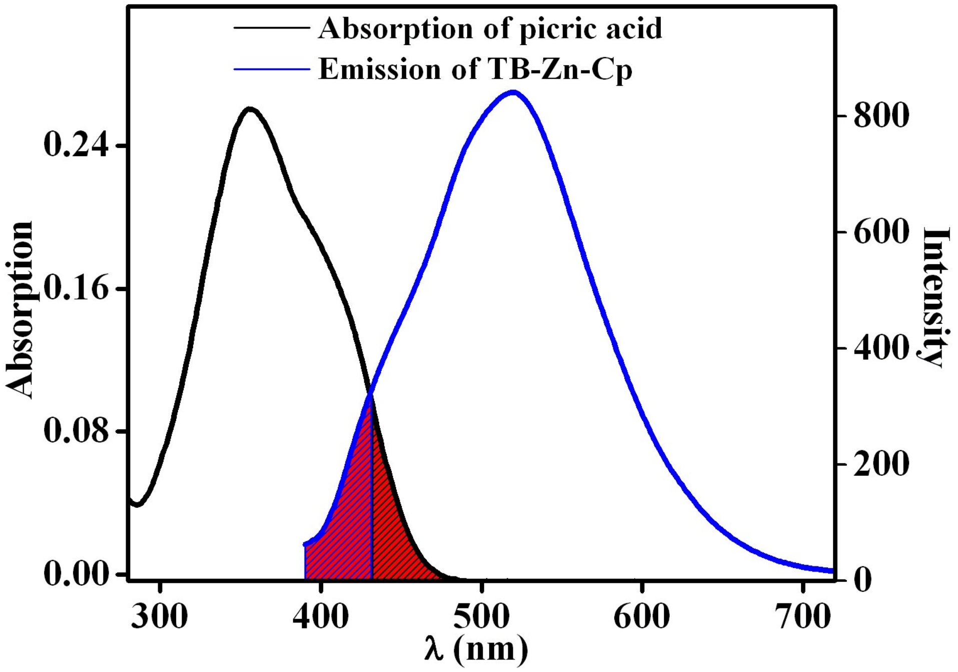

Fig. S25. The comparison of the absorption spectrum of PA with the emission spectrum of **TB-Zn-CP**.

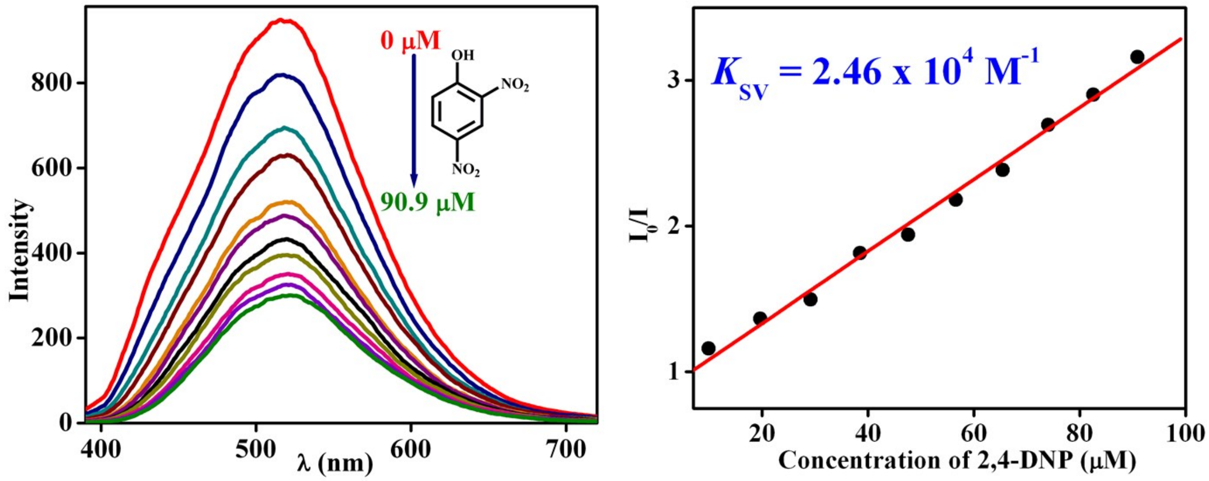

Fig. S26. Observed fluorescence quenching (left) of **TB-Zn-CP** upon addition of 2,4-DNP (μM) in water and corresponding Stern–Volmer plot (right).

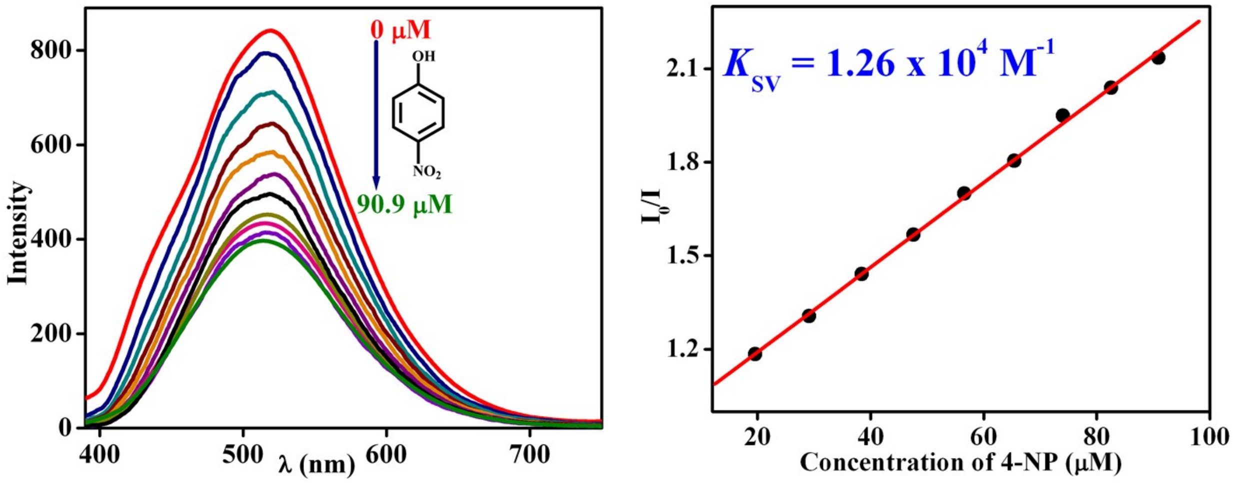

Fig. S27. Observed fluorescence quenching (left) of **TB-Zn-CP** upon addition of 4-NP (μM) in water and corresponding Stern–Volmer plot (right).

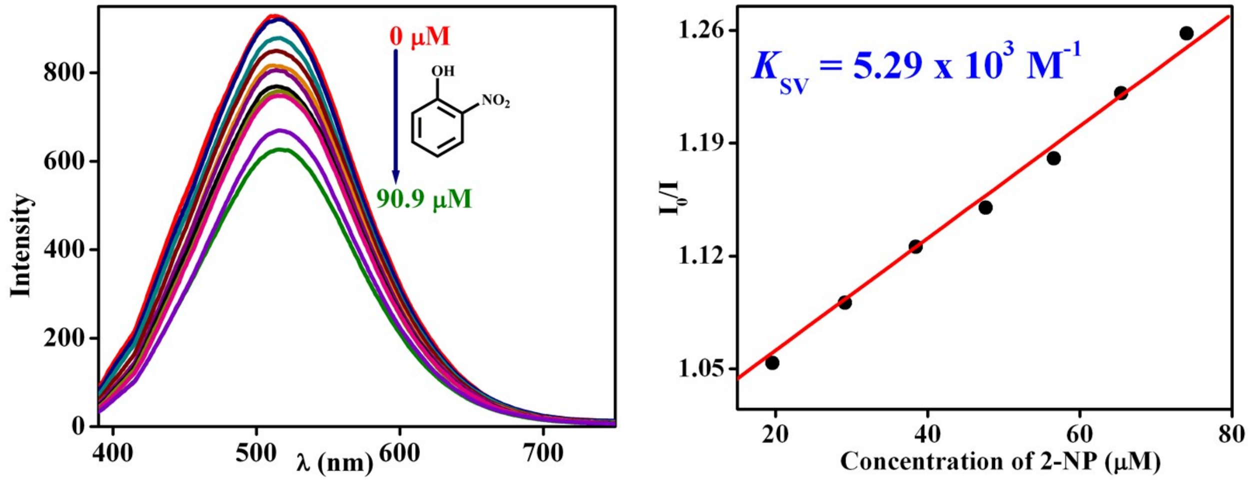

Fig. S28. Observed fluorescence quenching (left) of **TB-Zn-CP** upon addition of 2-NP (μM) in water and corresponding Stern–Volmer plot (right).

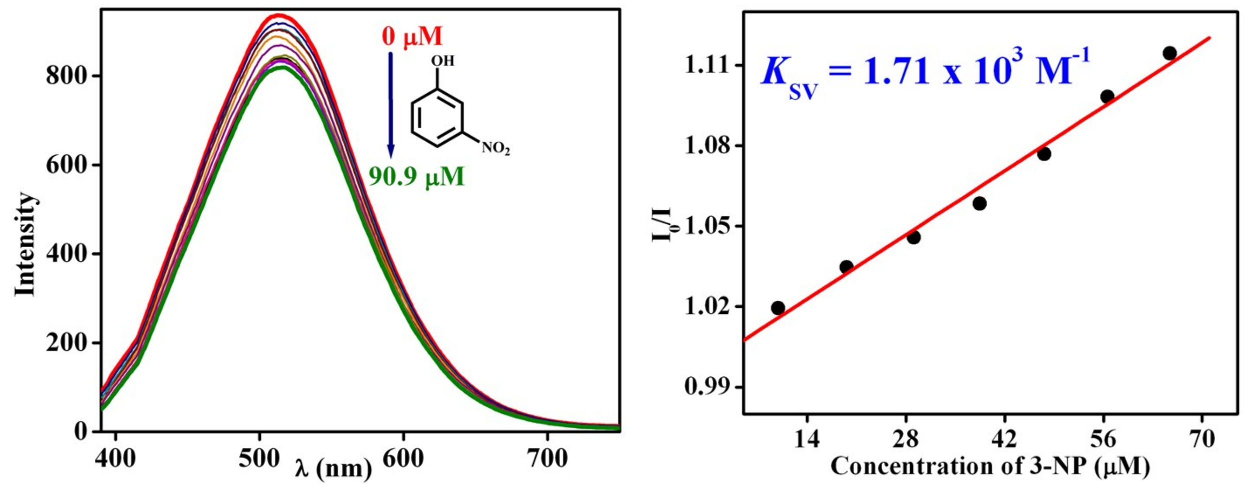

Fig. S29. Observed fluorescence quenching (left) of **TB-Zn-CP** upon addition of 3-NP (μM) in water and corresponding Stern–Volmer plot (right).

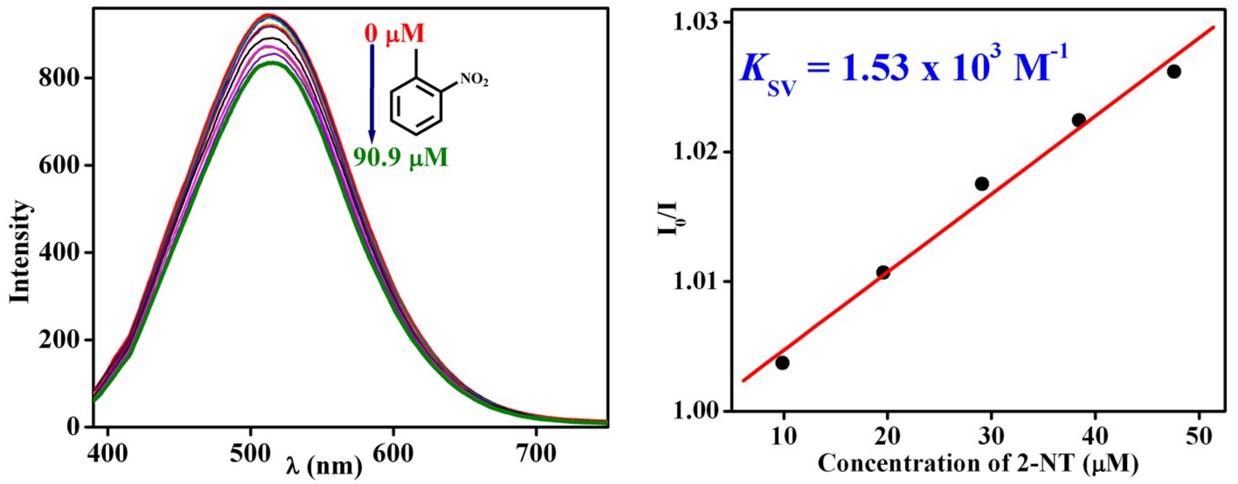

Fig. S30. Observed fluorescence quenching (left) of **TB-Zn-CP** upon addition of 2-NT (μM) in water and corresponding Stern–Volmer plot (right).

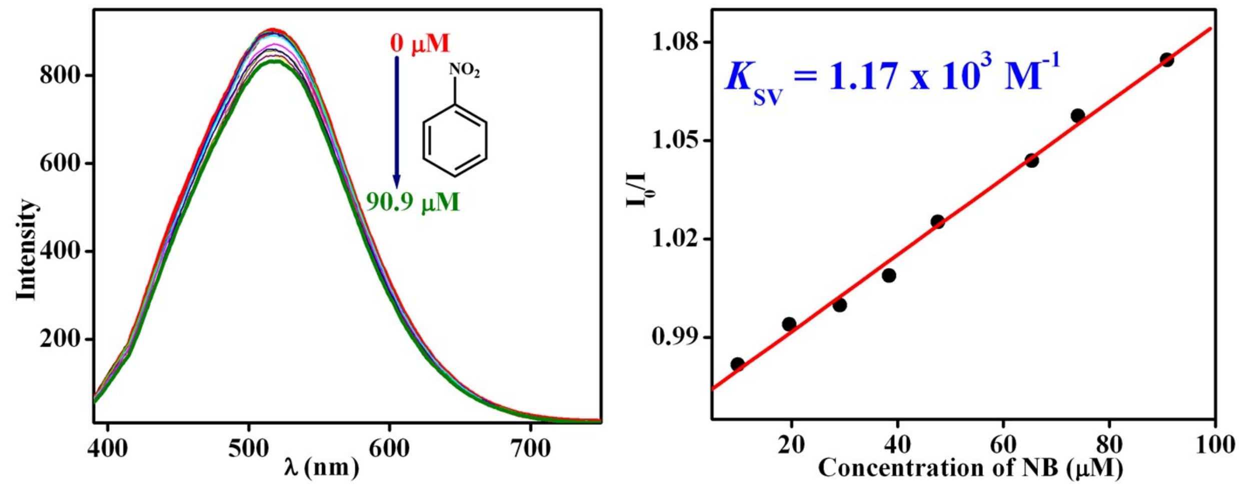

Fig. S31. Observed fluorescence quenching (left) of **TB-Zn-CP** upon addition of NB (μM) in water and corresponding Stern–Volmer plot (right).

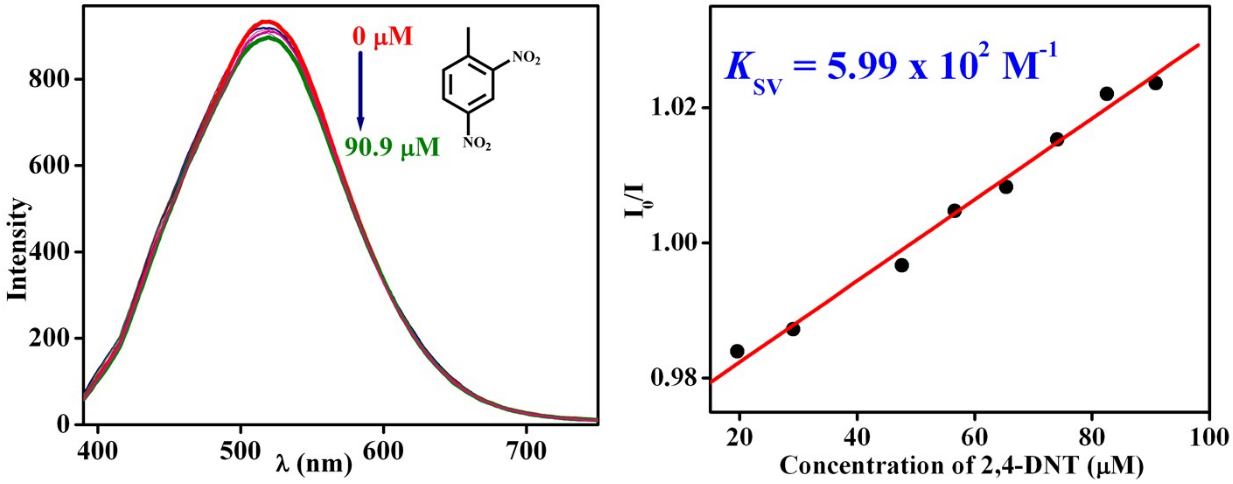

Fig. S32. Observed fluorescence quenching (left) of **TB-Zn-CP** upon addition of 2,4-DNT (μM) in water and corresponding Stern–Volmer plot (right).

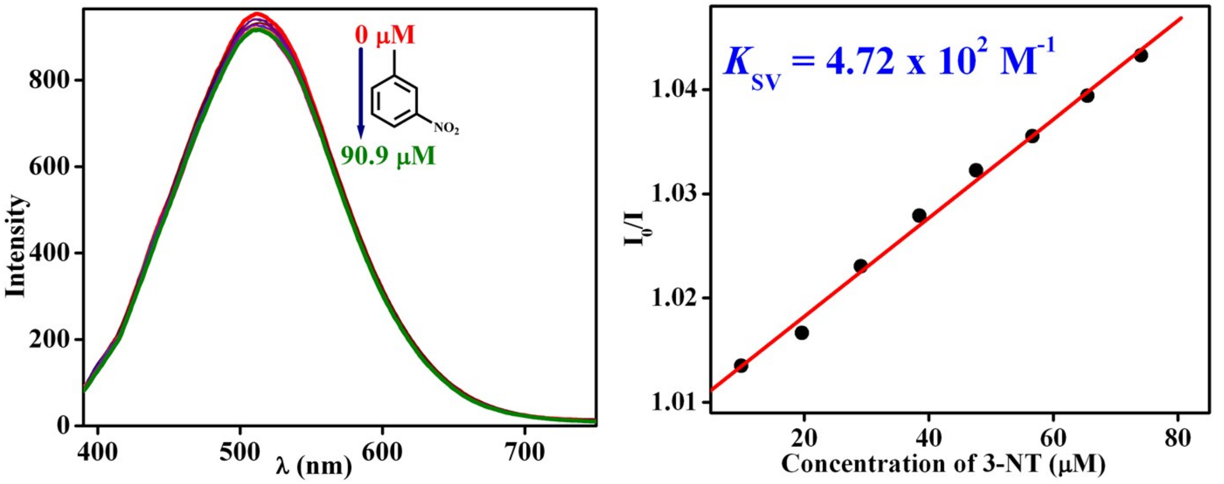

Fig. S33. Observed fluorescence quenching (left) of **TB-Zn-CP** upon addition of 3-NT (μM) in water and corresponding Stern–Volmer plot (right).

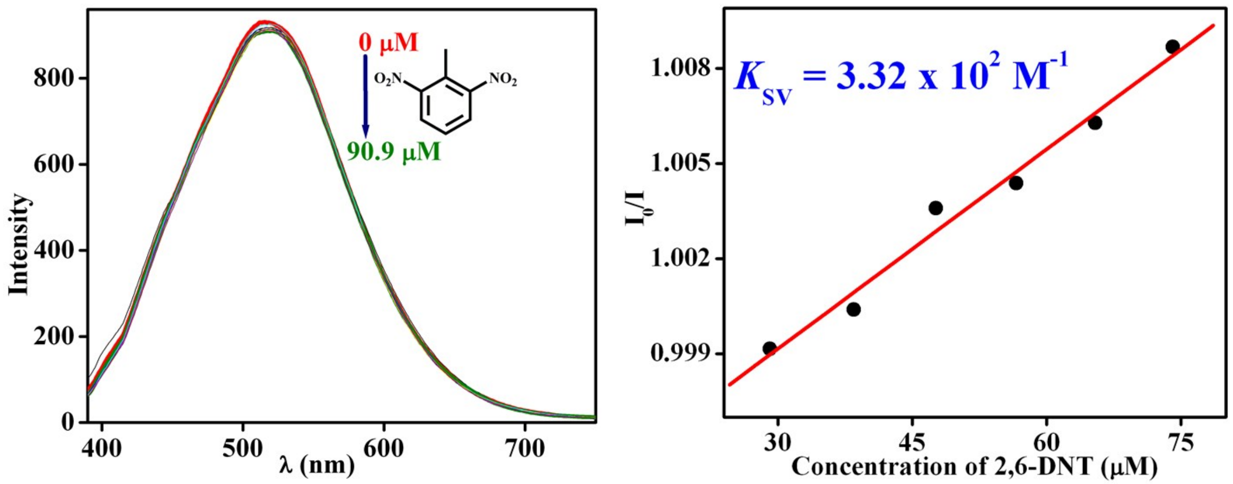

Fig. S34. Observed fluorescence quenching (left) of **TB-Zn-CP** upon addition of 2,6-DNT (μM) in water and corresponding Stern–Volmer plot (right).

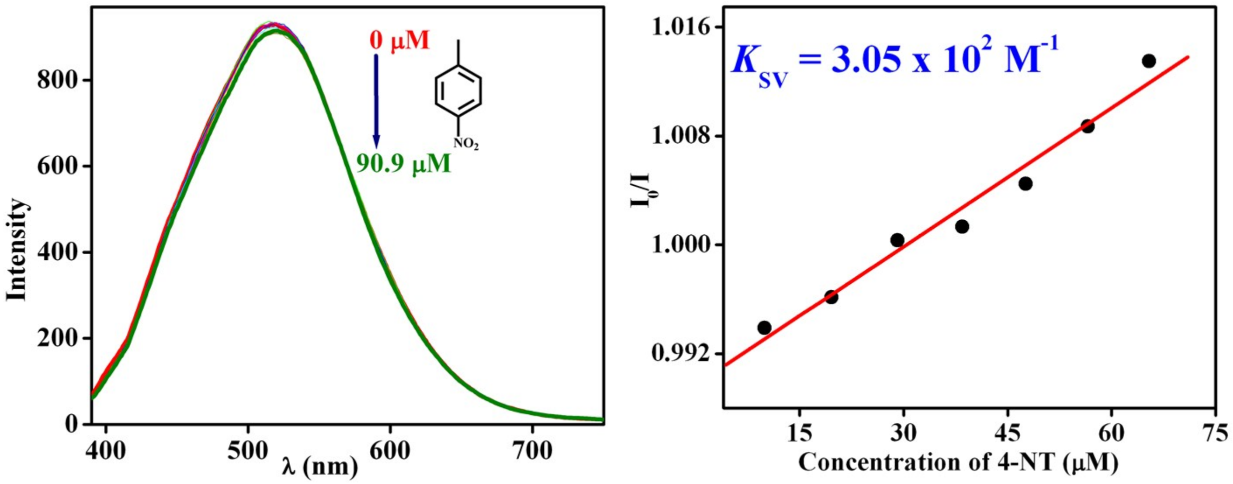

Fig. S35. Observed fluorescence quenching (left) of **TB-Zn-CP** upon addition of 4-NT (μM) in water and corresponding Stern–Volmer plot (right).
